# Simultaneous Quantification of L-Arginine and Monosaccharides during Fermentation: An Advanced Chromatography Approach

**DOI:** 10.3390/molecules24040802

**Published:** 2019-02-22

**Authors:** Mireille Ginésy, Josefine Enman, Daniela Rusanova-Naydenova, Ulrika Rova

**Affiliations:** Biochemical Process Engineering, Division of Chemical Engineering, Department of Civil, Environmental and Natural Resources Engineering, Luleå University of Technology, SE-971 87 Luleå, Sweden; mireille.ginesy@gmail.com (M.G.); droussanova@hotmail.com (D.R.-N.); ulrika.rova@ltu.se (U.R.)

**Keywords:** L-arginine, monosaccharides, ion chromatography, reversed-phase liquid chromatography, method validation, integrated pulsed amperometric detection, charged aerosol detector, fermentation

## Abstract

Increasing demand for L-arginine by the food and pharmaceutical industries has sparked the search for sustainable ways of producing it. Microbial fermentation offers a suitable alternative; however, monitoring of arginine production and carbon source uptake during fermentation, requires simple and reliable quantitative methods compatible with the fermentation medium. Two methods for the simultaneous quantification of arginine and glucose or xylose are described here: high-performance anion-exchange chromatography coupled to integrated pulsed amperometric detection (HPAEC-IPAD) and reversed-phase ultra-high-performance liquid chromatography combined with charged aerosol detection (RP-UHPLC-CAD). Both were thoroughly validated in a lysogeny broth, a minimal medium, and a complex medium containing corn steep liquor. HPAEC-IPAD displayed an excellent specificity, accuracy, and precision for arginine, glucose, and xylose in minimal medium and lysogeny broth, whereas specificity and accuracy for arginine were somewhat lower in medium containing corn steep liquor. RP-UHPLC-CAD exhibited high accuracy and precision, and enabled successful monitoring of arginine and glucose or xylose in all media. The present study describes the first successful application of the above chromatographic methods for the determination and monitoring of L-arginine amounts during its fermentative production by a genetically modified *Escherichia coli* strain cultivated in various growth media.

## 1. Introduction

L-arginine (hereafter referred to as arginine) is an important dietary supplement in animal feed [1,2,3] and has several potential clinical applications, such as in the treatment or prevention of cardiovascular diseases, diabetes type II, Alzheimer’s disease or infertility [4,5,6]. It also promotes wound healing [7] and is a key intermediate in the urea cycle. Arginine is one of the main amino acids in grapes and wines; however, its degradation can lead to the formation of ethyl carbamate, a possible human carcinogen [8,9,10]. An increased demand for arginine has driven the search for a more sustainable production that replaces keratin hydrolysis [11]. This has led to the use of *Corynebacterium glutamicum* and *Corynebacterium crenatum* for the fermentative production of arginine [11,12]. Unlike members of the *Corynebacterium* genus, *Escherichia coli* can metabolize five-carbon sugars, making it another potential candidate for arginine production, even if at lower yields [13]. 

Most methods for arginine analysis have focused on its quantification in drugs [14], biological samples such as urine or plasma [15,16], animal feed [17], grape juice, and wine [18,19] or to determine the amino acid composition of foods [20,21]. To monitor and optimize its microbiological production, fast and reliable methods for the analysis of arginine in fermentation samples are required. Considering that product formation and substrate consumption are both critical parameters in fermentation processes, a method that enables simultaneous quantification of arginine and sugars would be advantageous. 

Existing methods for arginine quantification have relied on either colorimetric or enzymatic reactions [22,23,24,25]. Although relatively fast and inexpensive, these methods are not suitable for fermentation samples, whereby ammonia, a common nitrogen source for microorganisms, interferes with the analysis [25,26]. Direct chromatographic quantification of amino acids, including arginine, is impeded by the existence of similar structures and/or lack of suitable chromophores. Therefore, currently used analytical methods often include either pre- or post-column derivatization to yield derivatives detectable by fluorescence or UV spectroscopy. The most common methods for amino acid analysis are ion-exchange separation coupled with post-column derivatization with ninhydrin and pre-column derivatization with a variety of reagents followed by reversed-phase high-performance liquid chromatography. The latter is arguably more sensitive, but is also limited by extensive sample manipulation, unstable derivatives, excess reagents and, in some cases, interference with other components present in the sample [27,28,29,30]. 

Recently, a few methods for the analysis of non-derivatized amino acids have been proposed. Some involve tandem mass spectrometry coupled with either liquid chromatography or hydrophilic interaction liquid chromatography (HILIC) [31,32], as well as the combination of HILIC with charged aerosol detection (CAD). However, with this method arginine appears to co-elute with histidine [33]. Combinations of reversed-phase ultra-high-performance liquid chromatography (RP-UHPLC) and CAD have also been proposed for amino acids detection, although they have not been fully validated [34,35]. Amino acid quantification using CAD has been tested with protein samples [33,35], but not with fermentation samples. To the best of our knowledge, CAD-detection has not been applied for the simultaneous quantification of amino acids and monosaccharides in fermentation broths, although sugars alone have been detected in this way [36,37,38].

High-performance anion-exchange chromatography with integrated pulsed amperometric detection (HPAEC-IPAD) has been shown to enable separation and detection of amino acids, carbohydrates, alditols, and glycols without the need for derivatization [39,40]. Due to its simplicity and the possibility to simultaneously detect arginine and carbohydrates, it is a promising method for the analysis of fermentation samples. So far, the method has been applied for the determination of initial amino acid content in various standard cell cultivation media, but not for their detection during fermentation [39,40,41] or fermentative production of arginine. 

In this study, HPAEC-IPAD and RP-UHPLC-CAD were fully validated for simultaneous analysis of arginine and glucose and xylose (as carbon sources) during fermentation. An existing HPAEC-IPAD method [40] was optimized and the suitability for arginine, glucose, and xylose quantification in commonly used fermentation media, i.e., lysogeny broth (LB), minimal medium (M9’), and complex medium containing corn steep liquor (CSL), was demonstrated. Corn steep liquor represents a readily available source of nitrogen, amino acids, vitamins, and other nutrients, commonly used in industrial processes, including the fermentative production of arginine [11,12,13]. In contrast, a defined medium is often preferred in the cosmetic and health-product industries to fully control media composition and the production process. Minimal medium is commonly used in studies targeting gene characterization or for strain development/selection [13,42,43,44]. Lysogeny broth is widely used in laboratories, although it is designed to work only at low bacterial densities [45,46]. In this study, the existing RP-UHPLC-CAD method [34] was re-designed and converted into a robust procedure for the thorough systematic quantification of arginine, glucose, and xylose in selected fermentation media. Subsequently, both methods were applied to follow sugar consumption and arginine production in actual fermentation samples during *E. coli* cultivation, and then compared in terms of performance and practicality. 

## 2. Results and Discussion

### 2.1. Method Optimization

#### 2.1.1. HPAEC-IPAD

The tailored short HPAEC-IPAD method uses a detection waveform recommended for amino acid analysis (“Gold, pH/Ag/AgCl RE, AAA”) and the reference electrode in pH mode [40]. The column and guard column were both maintained at 30 °C and injection volume was set to 25 µL. The flow rate was gradually increased, keeping the system pressure below the recommended limit during the entire gradient change (see Table 1), to a final flow rate of 0.75 mL/min. The procedure took 23 min in total, including the column regeneration step. Attempts to further shorten the procedure led to satisfying standard curves, but somewhat unstable retention times for glucose and xylose. 

**Figure 1:**
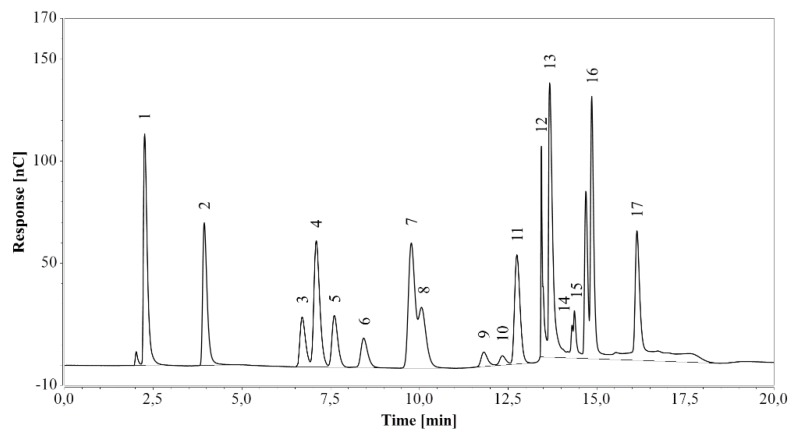
HPAEC-IPAD analysis of a standard amino acid mixture. 1—arginine, 2—lysine, 3—alanine, 4—threonine, 5—glycine, 6—valine, 7—serine, 8—proline, 9—isoleucine, 10—leucine, 11—methionine, 12—histidine/system peak, 13—phenylalanine, 14—glutamate, 15—aspartate, 16—cystine, 17—tyrosine.

The method was first applied on a standard amino acid mixture and all expected peaks were successfully identified in the chromatogram, although the histidine peak overlapped with the system peak (see Figure 1). All amino acids eluted at the expected times, within 17 min from the start. 

Analysis of a blank sample following injection of the standard mixture revealed no residual peaks. The same result was obtained with blank samples diluted 500 times and run after fermentation media analysis. As suggested by arginine, glucose, and xylose eluting at the corresponding retention times (see Section 2.2.3), the procedure achieved enough re-equilibration between each run.

However, under these conditions, arginine was poorly retained by the column and was eluted rather early together with similarly charged substances. Due to random inconsistencies, the influence of vial material was probed using pure water samples in different types of vials. A small peak at about 2.0 min was observed, yet it was sufficiently far from the peaks observed for arginine (2.25 min), glucose (7.26 min), and xylose (7.60 min) as to exclude any interference. However, vials or micro-inserts of glass generated a peak at 2.21 min, i.e. very close to the retention time of arginine. A persistent slightly negative signal was observed at the same retention time when polypropylene (PP) vials were used. 

**Figure 2:**
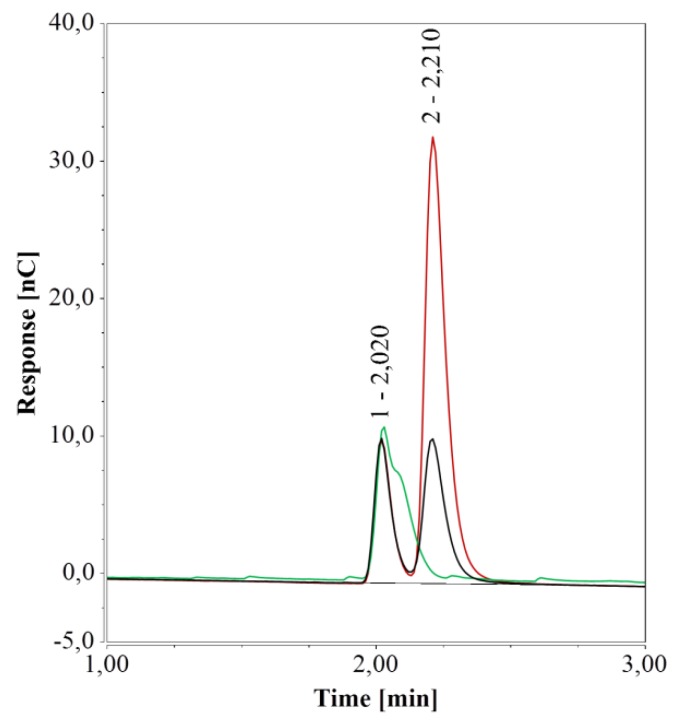
HPAEC-IPAD analyses of pure water samples in 1.5 mL glass vials; freshly prepared samples (black), samples stored for one week at 4 °C (red), and freshly prepared samples in a cleaned vial (green).

To further unravel this matter, sealed 1.5-mL glass vials and 0.3 mL PP vials containing pure water were kept at 4 °C for one week. The area of the peak at 2.21 min increased significantly over this period compared to that of freshly prepared water samples when glass vials were used (Figure 2) but remained unchanged for PP vials. It is worth noting that if arginine were present, the peak in the freshly prepared water sample in 1.5 mL glass vials would correspond to about 0.6 µmol/L of arginine and 2.4 µmol/L of arginine after one week. To overcome this undesired effect of glass vials, these were immersed in detergent for 12 h, thoroughly rinsed with pure water, and dried at 80 °C. As a result, the peak at 2.21 min was no longer detected in fresh water samples (Figure 2). 

Given that the peak area increased when water was kept in the glass vials but could not be detected neither with PP vials nor with cleaned glass vials, it appeared to be caused by interaction(s) between water and the surface of the manufactured vials.

To probe the effect of this additional peak on arginine quantification, duplicate samples with 10.00 µmol/L of arginine in 1.5 mL glass vials were analyzed and data were compared to arginine standard samples analyzed in 0.3 mL PP vials. Analysis revealed an arginine concentration of 10.55 µmol/L in the 1.5 mL glass vials. The effect of glass vials was more pronounced for samples with lower arginine concentrations and/or those not analyzed directly after preparation. For example, samples containing 4.5 µmol/L of arginine yielded 7.1 µmol/L of arginine after one week at 4 °C. 

#### 2.1.2. RP-UHPLC-CAD

A major difference between the Corona ultra RS and the standard Corona Veo detector is that with the former the nebulizer temperature is adjustable between 5 °C and 100 °C, whereas with the latter it is limited to either 35 °C or 50 °C. Hence, the CAD nebulizer temperature was set to 35 °C, the column heated to 40 °C, and a gradient of 0.4% heptafluorobutyric acid (HFBA) and acetonitrile was selected as the mobile phase. The maximum flow rate was 0.45 mL/min. A 5-min re-equilibration step was added at the beginning of the gradient and the injection volume was 5 µL. Details of the gradient profile are listed in Table 2. The procedure was fully optimized to last 17 min overall, including column regeneration. 

**Figure 3:**
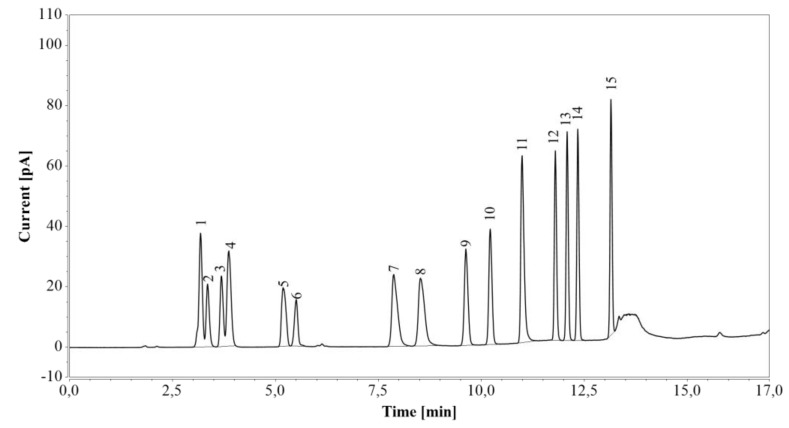
RP-UHPLC-CAD analysis of a standard amino acid mixture. 1—glycine/glutamic acid/serine, 2—aspartic acid, 3—threonine, 4 —alanine/glutamic acid, 5—proline, 6—cystine, 7—lysine, 8—histidine, 9—valine, 10—methionine, 11—arginine, 12—tyrosine, 13—isoleucine, 14—leucine, 15—phenylalanine.

First, RP-UHPLC-CAD was tested on a standard amino acid mixture (Figure 3). Arginine was completely separated from the other amino acids. Although glutamic acid eluted as two peaks (the first one together with glycine and serine, and the second one with alanine), the individual components of the mixture were fully identified, and their retention times confirmed by analysis of corresponding pure amino acid solutions. 

Again, no residual peaks were observed when blank samples were analyzed after injection of the standard amino acid mixture and various undiluted fermentation media. Together with the preservation of arginine, glucose, and xylose retention times (see Section 2.2.3), these results indicated that no other sample components were retained on the column and that column equilibrium was restored at the end of each gradient cycle.

Given the influence of vial material on HPAEC-IPAD analysis, its effect was investigated also for RP-UHPLC-CAD. Chromatograms generated from water samples in PP vials (0.3 and 0.7 mL) revealed a very small peak at about 3.1 min; this peak was much larger for samples in glass vials (0.1 and 1.5 mL) and was accompanied by a second peak at 2.125 min. Notably, both peaks’ areas were much larger for the 1.5-mL glass vials compared to the 0.1 mL micro-inserts. 

**Figure 4:**
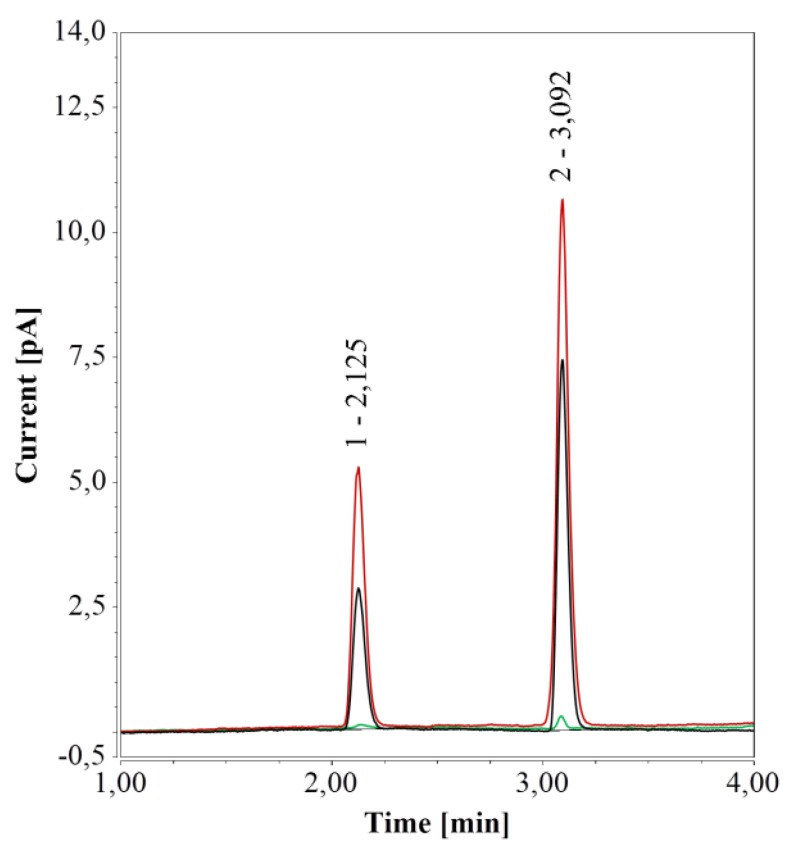
RP-UHPLC-CAD analyses of pure water samples in 1.5 mL glass vials; freshly prepared (black), after one week at 4 °C (red), and freshly prepared in a cleaned vial (green).

To further test the impact of different vial materials over time, water samples in either 0.7-mL PP vials or 1.5 mL glass vials were kept at 4 °C for one week and then analyzed. For samples in PP vials, RP-UHPLC-CAD chromatograms remained unchanged over time, compared to freshly prepared samples. However, for samples in glass vials, the areas of the two peaks increased considerably over time, in line with a previous study on ultrapure and pure water samples stored in glass bottles and analyzed by CAD [47]. When glass vials were cleaned (see Section 2.1.1), only a small peak at 3.1 min, similar to the peak in PP vials, remained (see Figure 4). 

With HPAEC-IPAD, the peak caused by glass vials overlapped with that of arginine. With RP-UHPLC-CAD, the first peak had a retention time (2.125 min) very close to those of glucose (2.178 min) and xylose (2.217 min). To examine whether this peak would interfere with the analysis, glucose or xylose solutions (12.5 mg/L) in 1.5-mL glass vials were analyzed directly or after one week of storage at 4 °C. Glass vials had no effect on glucose quantification in fresh samples and one-week storage had only a marginal influence; analytical determination of xylose was not affected at all (data not shown). Thus, glass vials were deemed compatible with this method.

### 2.2. Method Validation

Validation of the HPAEC-IPAD and RP-UHPLC-CAD methods followed standard procedures [48,49]. First, the suitability of the systems and their specificity towards arginine, glucose, and xylose quantification were verified. The calibration type, range, limit of detection (LOD), and limit of quantification (LOQ) were determined for each analyte. Accuracy and precision of the methods were confirmed in different fermentation media, for each analyte individually and for binary mixtures of arginine/glucose or arginine/xylose. 

#### 2.2.1. Suitability of HPAEC-IPAD

To verify the effectiveness of the operating system, a suitability test was conducted, whereby a series of replicate injections (*n* = 6) were examined for each analyte. Relative standard deviations (RSDs) of peak area measurements and retention times were <2% (Table 3), confirming the suitability of the system [48]. 

#### 2.2.2. Suitability of RP-UHPLC-CAD

Suitability testing of RP-UHPLC-CAD indicated that all RSDs of retention times were <0.3% (Table 4). RSDs of peak area measurements were 1.21%, 0.66%, and 1.13% for glucose, xylose, and arginine, respectively. Given that all RSD were well <3%, this operating system was deemed highly suitable [48]. 

#### 2.2.3. Specificity of HPAEC-IPAD

Specificity of the method was assessed by comparing the retention time of each analyte in a mixed sample to those in individual standard solutions [48]. 

Retention times of arginine, glucose, and xylose at different concentrations in M9’, LB, and CSL media were compared (see Table 5). The retention time of arginine in a standard amino acid mixture was also probed. The retention times for samples in the ranges of 0.125–20 mg/L (for glucose and xylose) and 0.50–90 µmol/L (for arginine) in pure water, are listed in supporting information (Table S1). Regardless of medium and/or concentration, RSDs between retention times were <3% for all analytes, indicating very good specificity of the method [48].

It should be noted that other compounds contained in fermentation samples, such as by-products or medium components may still elute at the same time as arginine, glucose or xylose and thus interfere with their analysis. Thus, each medium (M9’, LB, and CSL) was analyzed separately by HPAEC-IPAD. No additional M9’ components were detected around the retention times of arginine (2.25 min), glucose (7.26 min), and xylose (7.60 min). 

Analysis of lysogeny broth medium revealed a peak with identical retention time as arginine, corresponding to 1.82 ± 0.04 mmol/L of arginine in undiluted LB. This amount is in line with previous reports of 2–3 mmol/L arginine in LB medium [46]. A certain variation is expected given that LB contains yeast extract and tryptone, which result in a variable composition of complex components [50,51]. A minor peak at the retention time of glucose, most likely resulting from traces of sugars in the medium [46], was also detected. Importantly, analysis of spiked LB samples showed that glucose quantification was not affected (see Section 2.2.7). 

Analysis of corn steep liquor medium by HPAEC-IPAD revealed the presence of a peak (2.21 min) near the retention time of arginine, corresponding to 7.14 mmol/L in undiluted medium. This finding is not unexpected, given the mixture of soluble proteins, amino acids, carbohydrates, organic acids, vitamins, and minerals constituting CSL. Accordingly, substances similar to arginine, as well as arginine itself, could elute at the same time. Small amounts of arginine have been reported in CSL [52,53]. The observed peak was clearly related to CSL composition as all other components were present also in neat M9’medium, in which no such peak was detected. Another low-intensity peak, near the retention time for glucose, was observed and was related to the presence of glucose in CSL [54]; however, its concentrations was too low to be quantified when samples were diluted 1000 times. 

#### 2.2.4. Specificity of RP-UHPLC-CAD

RP-UHPLC-CAD specificity [48] was first determined by comparing the retention time of each analyte in water, M9’, LB, and CSL medium (Table 6). Arginine retention time was established using both an arginine standard and a standard amino acid mixture. Several concentrations were screened and retention times for additional concentrations in water are available in supporting information (Table S2). Glucose and xylose appeared to elute at similar times, i.e., around 2.2 min, indicating that the method could be used only if glucose and xylose served as separate substrates for arginine production or if their sum was of interest. The RSDs for RP-UHPLC-CAD retention times were <3%, irrespectively of medium and/or concentration, demonstrating very good specificity. 

To further assess the method’s specificity, retention times of by-products and/or media components were investigated to assess if they were close to those of the analytes of interest. All amino acids from the standard mixture were well separated from arginine and eluted after glucose and xylose (Figure 3) following application of a gradient. Therefore, any amino acid present in the fermentation medium or produced during cultivation would not be expected to interfere with the quantitative analysis. 

A similar analysis of the media (M9’, LB, and CSL) revealed no peaks around the retention time of arginine (near 11.0 min) in M9’ and CSL. However, as discussed in Section 2.2.3, the arginine peak observed in LB medium by HPAEC-IPAD, was detected also by RP-UHPLC-CAD, where it amounted to 1.82 ± 0.11 mmol/L of arginine in undiluted LB. This observation confirmed the good specificity of the method for arginine.

Analysis of M9’medium did not show additional peaks at/around glucose or xylose retention times, while in case of LB and CSL, a few peaks appeared between 2.1 min and 2.3 min. These peaks were found to impair analysis of low concentrations of glucose in LB and CSL and of xylose in CSL medium (see Section 2.2.8). Their existence likely related to traces of carbohydrates in LB and CSL media [46,54].

#### 2.2.5. Reportable Range and Calibration Type for HPAEC-IPAD

The range over which the test could be performed was evaluated by diluting standard stock solutions of arginine, glucose, and xylose. Area and height of the peaks were suitable for analysis of all three analytes and, hereafter, the area was always chosen as the evaluation parameter. 

**Figure 5:**
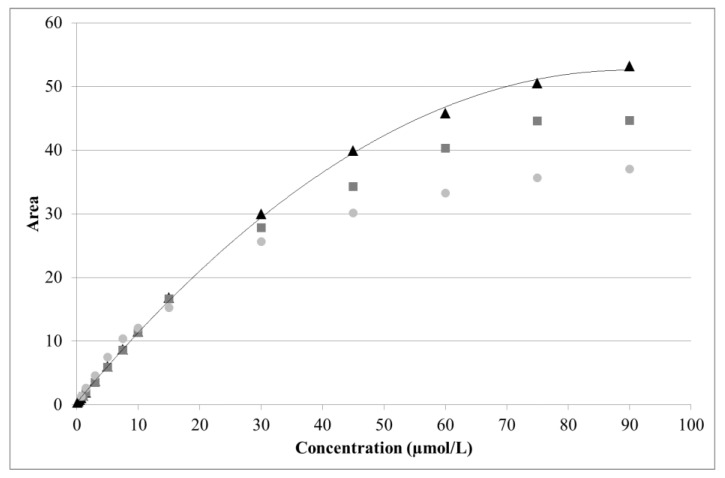
Effect of electrode aging on arginine analysis. Three series of standards were analyzed using the same electrode after 0 h (black triangles), 36 h (dark gray squares), and 48 h (light gray circles) of continuous use. Solid line: quadratic fitting curve.

For arginine, a near linear response was obtained from 0.5 to 15 µmol/L (R^2^ = 0.9988). A quadratic curve with offset provided a good fit over a much wider range (1.5–90 µmol/L). However, due to aging of the disposable gold electrode, detector response was seen to decrease over time (Figure 5). As the latter was particularly pronounced above 30 µmol/L of arginine, the useful range was restricted to 1.5–30 µmol/L. 

The dynamic range, evaluation parameter, and calibration type chosen for each analyte are summarized in Table 7, along with an example of the y-intercept, slope, curvature, and coefficient of determination (R^2^). The corresponding calibration plots are provided in supporting information (Figures S1–S3). R^2^ was always > 0.997 and the y-intercept was zero within the 95% confidence interval. A linear fit was acceptable for both glucose (2.0–20.0 mg/L) and xylose (1.0–20.0 mg/L). Although less noticeable than for arginine, the signals from glucose and xylose were seen to weaken due to electrode aging (Figure S2). 

It should be noted that the decline in detector response, together with small variations from electrode to electrode (Figures S1–S3), implies that new calibration standards must be regularly tested between sets of samples to ensure adequate data accuracy. 

#### 2.2.6. Evaluation Parameter, Calibration, and Range for RP-UHPLC-CAD

Calibration curves were established by analyzing triplicate series of standard solutions. Evaluation and calibration types, y-intercept, slope, curvature, and R^2^ of the calibration curves, as well as the range are described in Table 8. The corresponding calibration plots are available in supporting information (Figures S4–S6).

Quadratic curves with offset, calculated by the method of least squares, could be fitted to the responses. Although both height and area resulted in reasonably good fits (*R^2^* > 0.9985), area provided greater accuracy for all three analytes. *R^2^* was then always > 0.999 and the y-intercept was zero within the 95% confidence interval. For all analytes, the range covered three orders of magnitude (Table 8), which gives important advantages. First, it is easier to dilute the samples to an appropriate level even when the analyte(s) concentration is unknown beforehand. Second, it is more likely that arginine and glucose or xylose can be quantified with a single dilution, even when present at very different concentrations.

To investigate calibration curves stability, identical series of standards were analyzed at different times. For all analytes, the difference between the standard points was minimal (Figures S4–S6), indicating that the same standard curves could be used over a prolonged time. 

#### 2.2.7. Accuracy of HPAEC-IPAD

A method is considered accurate when the analytical result is within 90–110% of either a conventional true value or an accepted reference value [48]. One way of measuring accuracy is to spike the analyte in blank matrices and compare the obtained value with the expected one. 

As the objective of this study was to establish the suitability of this method for fermentation samples, accuracy was tested in diluted M9’, LB, and CSL media. The results are reported as percentage recovery of the known added amount of arginine (Table 9) or glucose and xylose (Table 10). 

In M9’, arginine recovery was within 96–102% of the expected value for the three concentrations tested. Considering the presence of arginine in LB medium (see Section 2.2.3), 1.82 µmol/L of arginine were added to the expected value of 1000 times diluted medium, resulting in a recovery of 96–103%. Recovery of arginine in M9’ and LB was thus always within the acceptable 90–110% range [48], including when the media were spiked with a standard amino acid mixture instead of pure arginine. 

As anticipated, CSL medium spiked with arginine always resulted in a higher concentration (7.61 ± 0.13 µmol/L) than the expected one (7.14 ± 0.04 µmol/L in 1000 times diluted medium). It should be emphasized that in real fermentation samples, arginine concentration cannot be corrected by subtracting this value. Indeed, the latter might be caused by component(s) consumed by bacteria, meaning that the amount would change throughout the fermentation. 

In all media, the recovery for glucose and xylose was 99–103% and 98–105%, respectively. Therefore, except for arginine analysis in CSL, the proposed method is sufficiently accurate in these media.

#### 2.2.8. Accuracy of RP-UHPLC-CAD

Accuracy was evaluated by spiking each analyte in 100 times diluted media and comparing triplicates of three different concentrations [49]. In each medium, arginine recovery was within the acceptance range, even when media were spiked with a standard amino acid mixture instead of pure arginine (Table 11). In the case of LB medium, 0.0182 mmol/L arginine was added to the expected value for 100 times diluted medium. Glucose and xylose could be quantified with good accuracy in M9’ medium (Table 12). However, recovery of glucose at a low concentration (0.01 g/L) in LB and CSL, as well as of xylose in CSL was affected by the presence of interfering components in these media. At higher concentrations (>0.05 g/L), interferences became negligible and recovery was 90–110%.

#### 2.2.9. Precision of HPAEC-IPAD

Precision is defined as the closeness between analytical results obtained from multiple analyses of the same homogeneous sample. It comprises three different levels: repeatability, intermediate precision, and reproducibility. The latter, representing the precision between laboratories, was beyond the scope of this study.

Repeatability describes precision under the same operating conditions over a short interval of time. In this study, repeatability was evaluated in all media by consecutively analyzing triplicates of the same samples at three different concentrations [49]. Relative standard deviations for each sample are presented in Table 9 and Table 10.

Intermediate precision is an evaluation of variations within a laboratory. Here, it was examined by repeating the same analytical procedure for triplicates of the same samples on three different days. CSL medium, diluted 1000 times, was spiked with arginine (15 µmol/L), glucose (7.0 mg/L) or xylose (7.0 mg/L). Mean, standard deviations, and RSDs are presented in Table 13. As all RSDs were <10%, intermediate precision was deemed acceptable [48] providing that any existing amount of arginine in the medium and electrode aging were accounted for.

#### 2.2.10. Precision of RP-UHPLC-CAD

For RP-UHPLC-CAD, precision was assessed based on repeatability and intermediate precision. Repeatability was evaluated by analyzing triplicates of samples at three concentrations in the three different media. All RSDs were <5% (Table 11 and Table 12), demonstrating good repeatability of the method.

Intermediate precision was studied in CSL medium diluted 100 times and spiked with arginine (0.75 mmol/L), glucose (0.100 g/L) or xylose (0.100 g/L). Analyses of triplicates of each sample were repeated on three different days. As all RSDs were <2% (Table 14), intermediate precision was deemed very good (i.e., greatly < 10%) [48,49]. 

#### 2.2.11. Limit of Detection and Limit of Quantification for HPAEC-IPAD

The LOD of an analyte represents the lowest concentration that can be qualitatively detected. The LOQ represents the lowest concentration that can be determined with acceptable precision and accuracy. 

LOD and LOQ values were determined using samples with known, decreasing concentrations of analytes, at which a signal-to-noise ratio of 3 or 10 was obtained, respectively [49]. For arginine, the LOD was 0.1 µmol/L and the LOQ was 0.3 µmol/L (0.42 and 1.3 ng on the column, respectively). For glucose and xylose, the LOD was 30 µg/L (0.75 ng on the column) and 8 µg/L (0.2 ng on the column), respectively, whereas the LOQ was 1 mg/L (25 ng on the column) and 0.25 mg/L (6.3 ng on the column), respectively (Table 7). 

#### 2.2.12. Limit of Detection and Limit of Quantification for RP-UHPLC-CAD

Both limits were established in water by analyzing arginine, glucose, and xylose solutions with known, decreasing concentrations until the desired signal-to-noise ratios were obtained. For arginine, the LOD was 2 µmol/L (1.7 ng on the column) and the LOQ was 10 µmol/L (8.7 ng on the column). For glucose and xylose, the LOD was 0.3 mg/L (1.5 ng on the column) and 0.4 mg/L (2.5 ng on the column), respectively, whereas the LOQ was 1 mg/L for both (5 ng on the column) (Table 8).

#### 2.2.13. Binary Mixtures and Recovery Using HPAEC-IPAD

During fermentation, conditions gradually change from a high substrate/low product ratio to a low substrate/high product ratio. To represent different phases of the fermentation process, binary and ternary mixtures were prepared with different ratios between arginine and glucose and/or xylose concentrations. Samples were prepared in water, M9’, and LB. 

In binary mixtures, recovery was 96–102%, 96–105%, and 94–103% for arginine, glucose, and xylose, respectively (Table 15). Determination of a low concentration of an analyte was not affected by the presence of the other analyte at a high level.

**Figure 6:**
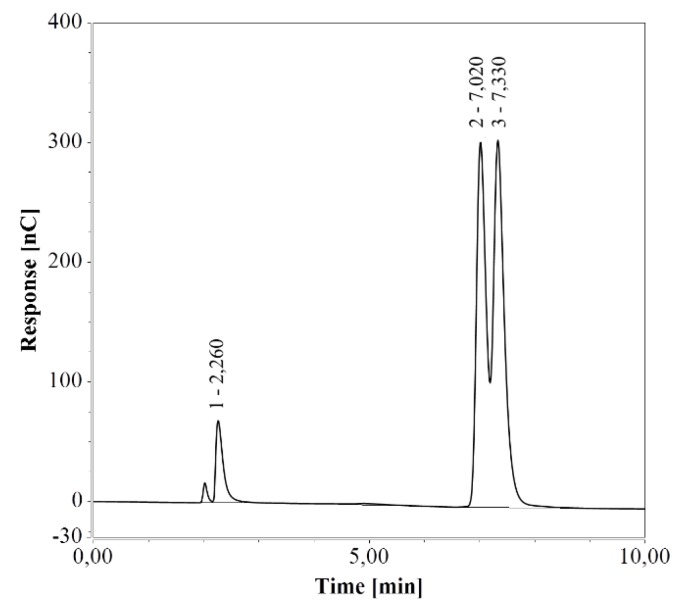
Ternary mixture separated by HPAEC-IPAD. 1 – arginine, 2 – glucose, 3 – xylose.

In ternary mixtures, the recovery was 98–101%, 95–101%, and 95–103% for arginine, glucose, and xylose, respectively (Table 16), confirming the suitability of the method even when mixtures of glucose and xylose were used as substrates. However, it should be noted that the glucose and xylose peaks were not completely separated (Figure 6) and further optimization of the method w.r.t. sodium hydroxide and sodium acetate concentration profiles will ensure better separation. 

#### 2.2.14. Binary Mixtures Separation Using RP-UHPLC-CAD

Model solutions in fermentation media, as well as a control in water, were prepared with various arginine/glucose and arginine/xylose ratios. The recoveries were all within the 90–110% range (Table 17), confirming that binary mixtures could be analyzed with satisfying accuracy even when one analyte was present at a low concentration and the other at a high amount.

### 2.3. Application to Fermentation Samples

**Figure 7:**
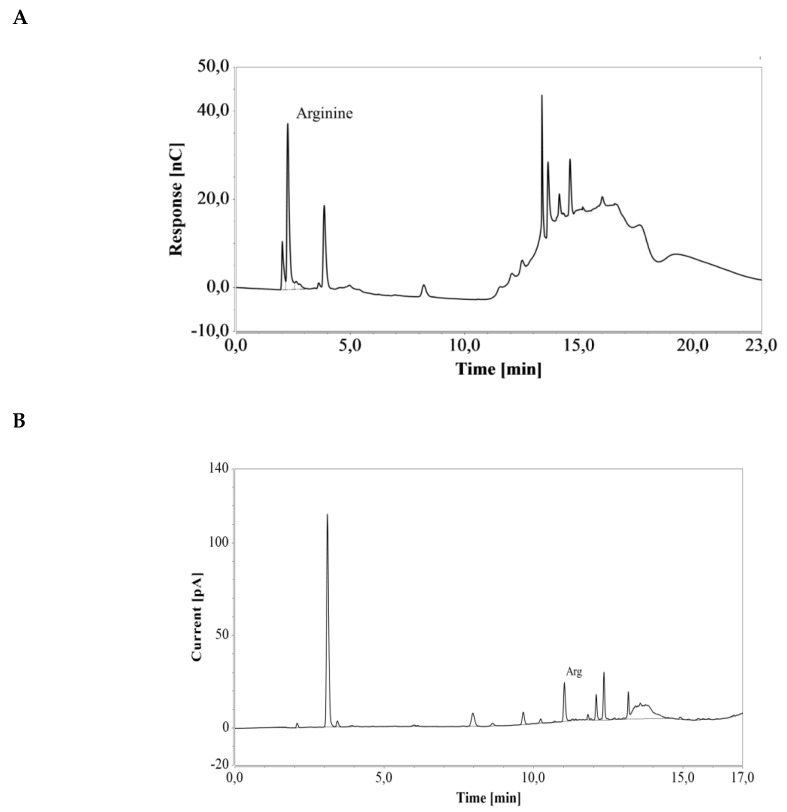
Chromatograms of a an *E. coli* shake flask culture after 40 h of cultivation in LB medium, based on (A) HPAEC-IPAD and (B) RP-UHPLC-CAD analyses. Unmarked peaks originate from media components.

*E. coli* cells engineered for enhanced arginine production were cultivated in a shake flask containing LB medium. Triplicate samples were taken after 40 h of cultivation and diluted 100 and 25 times for subsequent HPAEC-IPAD and RP-UHPLC-CAD analysis, respectively (see Figure 7). The resulting arginine concentration was 4.37 ± 0.07 mmol/L (0.76 ± 0.01 g/L) with the HPAEC-IPAD system and 4.47 ± 0.08 mmol/L (0.78 ± 0.01 g/L) with the RP-UHPLC-CAD system. The two values were in good agreement (RSD = 1.52%), suggesting that both methods offer excellent accuracy for the analysis of arginine produced during cultivation in LB medium.

**Figure 8:**
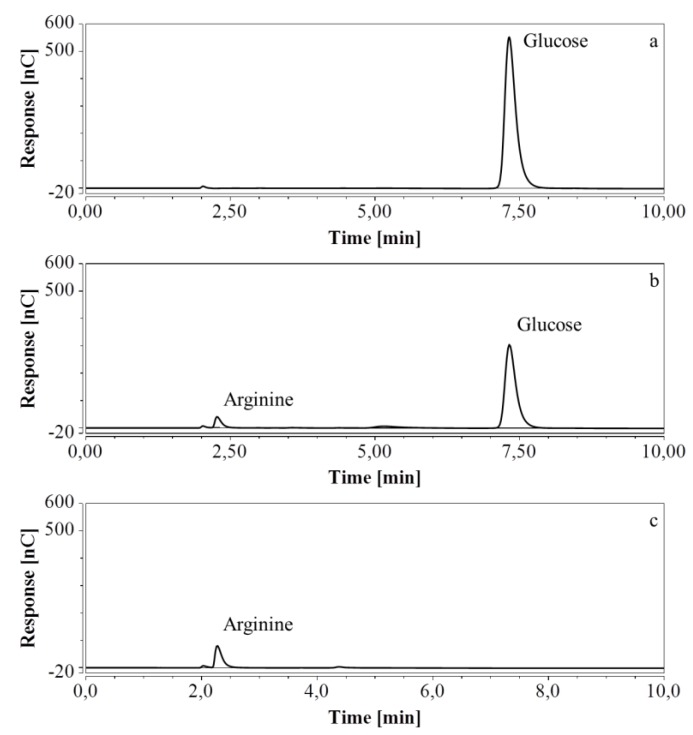
HPAEC-IPAD chromatograms of samples taken at various times of fermentation in M9’ with glucose as substrate (only the relevant part is shown). (**a**) t = 0 h, 2000 times dilution; (**b**) t = 24.25 h, 2000 times dilution; (**c**) t = 40.75 h, 1000 times dilution.

*E. coli* fermentations were also performed in bioreactors, using either M9’ medium with glucose or CSL medium with xylose. Triplicate samples taken throughout the fermentations were diluted so that the concentration of the analyte was within the range indicated in Table 7 and Table 8; when necessary, several dilutions were made. HPAEC-IPAD chromatograms of samples at the start and at the end of the fermentation process in M9’ with glucose as substrate exhibited a decrease in glucose but an increase in arginine over time (Figure 8). 

**Figure 9:**
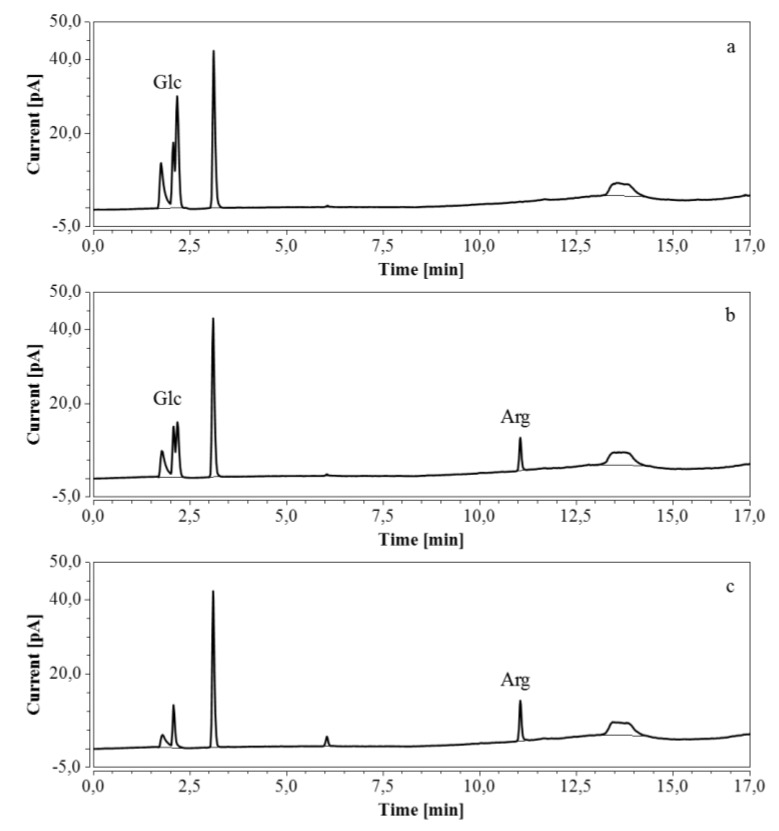
RP-UHPLC-CAD chromatograms of samples taken at various times of fermentation in M9’ with glucose as substrate. (**a**) t = 0 h, 100 times dilution; (**b**) t = 24.25 h, 100 times dilution; (**c**) t = 40.75 h, 100 times dilution. Unmarked peaks originate from medium components.

**Figure 10:**
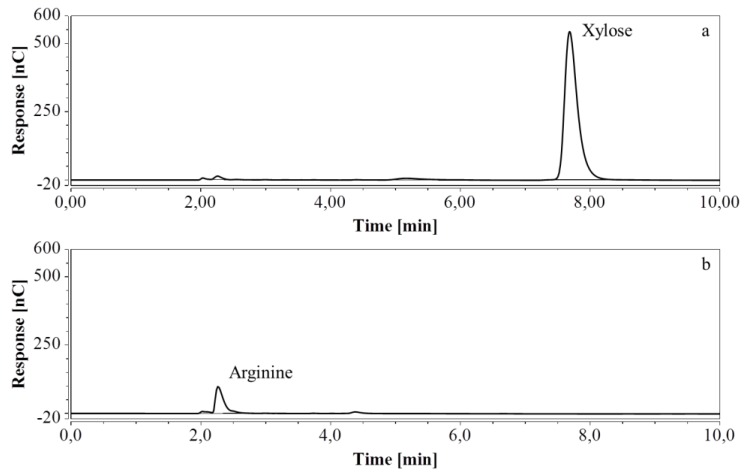
HPAEC-IPAD chromatograms of samples taken at various times of fermentation in CSL with xylose as substrate (only the relevant part is shown). (**a**) t = 0 h, 4000 times dilution; (**b**) t = 46.5 h, 2000 times dilution.

**Figure 11:**
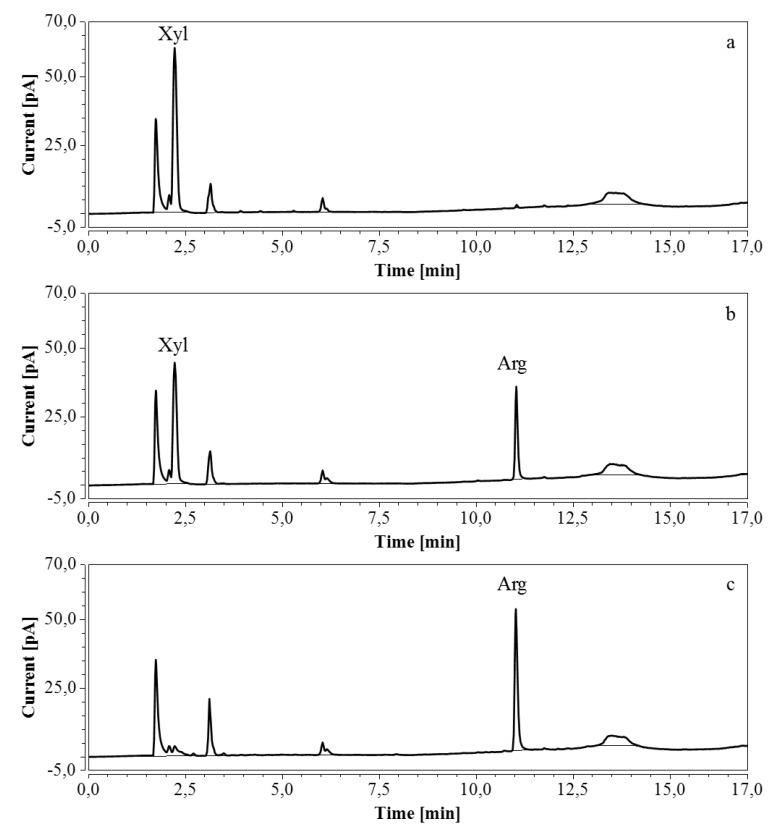
RP-UHPLC-CAD chromatograms of samples taken at various times of fermentation in CSL with xylose as substrate. (**a**) t = 0 h, 100 times dilution; (**b**) t = 31 h, 100 times dilution; (**c**) t = 46.5 h, 100 times dilution. Unmarked peaks originate from medium components.

For RP-UHPLC-CAD, the wide range of analysis (see Section 2.2.6) allowed a single dilution (approx. 100 times) to be applied to all samples for the determination of both arginine and the selected monosaccharides, at all fermentation stages. Representative chromatograms of samples taken at various times of the fermentations are shown in Figure 9 (M9’), as well as Figure 10 and Figure 11 (CSL). In all three cases an increasing amount of arginine, together with a decreasing concentration of glucose/xylose, was detected and quantitatively monitored by both HPAEC-IPAD and RP-UHPLC-CAD.

**Figure 12:**
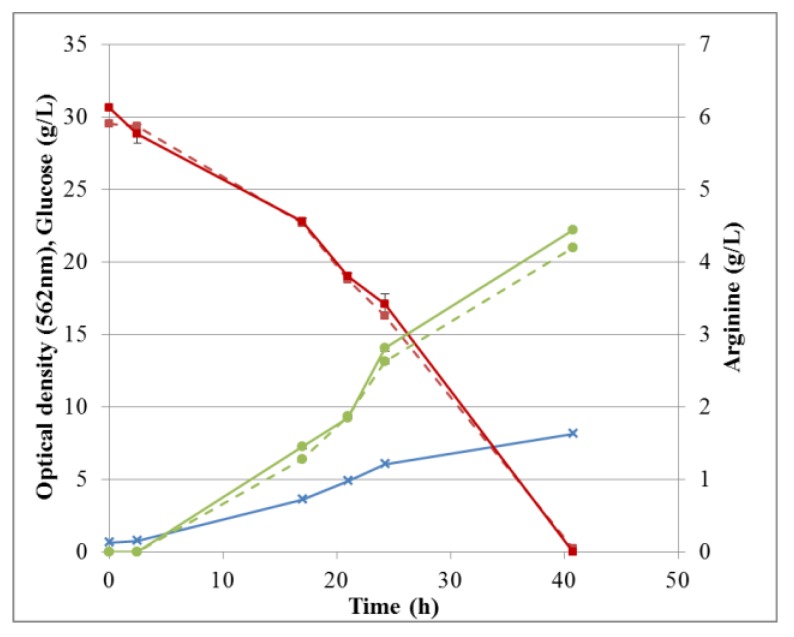
Fermentation profile: cell growth, glucose consumption, and arginine production in M9’. Optical density (blue crosses), glucose (red squares), and arginine (green circles) were determined by RP-UHPLC-CAD (solid lines) or HPAEC-IPAD (dashed lines). Standard deviations are omitted for clarity.

Overall arginine production and glucose consumption during fermentation in M9’ medium, together with the corresponding cell growth is shown in Figure 12. The corresponding data for CSL medium with xylose can be found in supporting information (Figure S7). Fermentation processes were followed by sampling at different times and the broths were analyzed independently by either HPAEC-IPAD or RP-UHPLC-CAD. Both methods were suitable for this purpose and the data were in excellent agreement. Specifically, for fermentation in M9’, the RSD between duplicates analyzed by HPAEC-IPAD was always <3.6% for both glucose and arginine, indicating a good precision of the method. The RSD between triplicates analyzed by RP-UHPLC-CAD was 0.5–2.5% for arginine and 0.7–3.9% for glucose. Thus, all RSDs were <5%, demonstrating very good repeatability of the method throughout the fermentation process. For all samples, the difference between results obtained by HPAEC-IPAD or RP-UHPLC-CAD was <2.6% for glucose and <8% for arginine, suggesting adequate accuracy. 

In CSL medium, RSDs between duplicates analyzed by HPAEC-IPAD were always <2.2% for xylose and <1.6% for arginine, again confirming good precision of the method. For analyses conducted by RP-UHPLC-CAD, RSDs were 0.2–4.0% for arginine and 0.01–0.1% for xylose. In M9’ fermentation samples, all RSDs were <5%. As described in Section 2.2.3, one or several compounds contained in CSL eluted at the same time as arginine using HPAEC-IPAD. Hence, during the first half of fermentation, arginine was noticeably higher when measured by HPAEC-IPAD than by RP-UHPLC-CAD. However, after 27 h, RSD between measurements with the two methods dropped <4%. This suggests that the component(s) interfering with arginine analysis was/were consumed during fermentation. Therefore, HPAEC-IPAD might be used for the determination of arginine during fermentation in CSL, depending on the accuracy needed during the early stage of the process. 

The difference between the two methods when quantifying xylose was minimal and RSDs were always <2.4%. Even though analysis of low xylose concentrations in CSL was impaired in model solutions, the last sample of a fermentation in CSL with xylose as substrate yielded nearly the same result with both methods (Figure 12). 

### 2.4. Comparison between HPAEC-IPAD and RP-UHPLC-CAD Systems

Both the RP-UHPLC-CAD and HPAEC-IPAD methods described here enabled the simultaneous quantification of arginine, glucose, and xylose. A comprehensive comparison of the main technical aspects of the two methods, their analytic characteristics, and the different parameters measured to assess their suitability, is provided in Table 18. The two methods displayed similar high repeatability and intermediate precision, with a somewhat higher accuracy for HPAEC-IPAD. 

The two analyses took approximately the same time: 22 min by RP-UHPLC-CAD and 23 min by HPAEC-IPAD. The effective time by HPAEC-IPAD might actually be longer, both because the start-up procedure is slightly more demanding than for RP-UHPLC-CAD and because a series of standard curve data points have to be regularly analyzed. With RP-UHPLC-CAD, the detector signal is stable over a longer time and standard curves can be used repeatedly. Importantly, both methods proposed in this study are considerably shorter than existing ones based on pre-column derivatization.

Regarding fermentation samples, the substrate and product concentrations can only be broadly estimated and for HPAEC-IPAD analysis, two different dilutions might be needed for samples containing a high concentration of one analyte and a low amount of the other(s). Instead, a single dilution is most likely enough to quantify both arginine and glucose or xylose using RP-UHPLC-CAD.

Furthermore, RP-UHPLC-CAD was validated in all tested media, whereas some component(s) of CSL co-eluted with arginine when using HPAEC-IPAD. Due to the specifics of analytical chromatography, arginine is retained longer on the reversed-phase column, while detectable salts and other media components seem to elute early. Vice versa, arginine has a very short retention time on the column used for HPAEC, as do also some CSL components that interfere with the analysis. Therefore, RP-UHPLC-CAD appears more suitable for arginine analysis when media such as CSL are used.

In contrast, HPAEC-IPAD delivered better specificity for glucose and xylose. The mixtures of glucose and xylose could be analyzed with satisfying accuracy, even though peak separation was not complete (this could be overcome by varying eluent concentrations). Nevertheless, this characteristic is a promising feature as a mixture of these sugars is a commonly used substrate for fermentation. Here, HPAEC-IPAD displayed a higher sensitivity for arginine than RP-UHPLC-CAD, which could offer a significant advantage when arginine is present only in trace amounts. 

In summary, both methods enable the quantification of arginine, glucose, and xylose in a robust, accurate, and precise way. Moreover, unlike HPAEC-IPAD, RP-UHPLC-CAD was validated in CSL, suggesting it might be generally better suited to fermentation sample analysis. In comparison, HPAEC-IPAD is a good option for fermentation samples in minimal medium with both glucose and xylose as substrates, or for samples whereby higher sensitivity is needed. 

## 3. Materials and Methods

### 3.1. Reagents, Solvents, Materials

Ultrapure water of high resistivity (18.2 MΩ, TOC < 4 ppm) generated using a Milli-Q purification system (Merck Millipore Advantage A10, Darmstadt, Germany) was used for preparation of samples and eluents. Spectroscopic grade 50–52% (w/w) sodium hydroxide solution and anhydrous sodium acetate was obtained from Sigma-Aldrich (Steinheim, Germany). HPLC-grade HFBA was purchased from Thermo Scientific (Rockford, IL, USA) and acetonitrile LiChrosolv® hypergrade for LC-MS was purchased from Merck (Darmstadt, Germany). For HPAEC-IPAD, the eluents were prepared as described previously [55] and thereafter blanketed under 1 psi of argon. 

Analytical standards for D-glucose (500 mg), D-xylose (500 mg), and arginine (100 mmol/L) were purchased from Sigma Aldrich (Buchs, Switzerland). An amino acid standard containing 2.5 µmol/L of 16 amino acids, including arginine, and 1.25 µmol/L of cystine was also obtained from Sigma Aldrich (St. Louis, MO, USA). 

Snap ring vials and caps for analysis were purchased from VWR (Langerwehe, Germany). Unless otherwise stated, analyses were conducted using transparent 0.3-mL PP vials with polytetrafluoroethylene caps. Transparent PP 0.7-mL vials, clear glass 1.5-mL vials, and clear glass 0.1-mL micro-inserts were also evaluated. 

High-purity grade chemicals were used to prepare culture media.

### 3.2. Sample Preparation

All data were obtained either in duplicates (*n* = 2) or triplicates (*n* = 3). For HPAEC-IPAD, all solutions were prepared with an accuracy of 0.005 mg/L; for RP-UHPLC-CAD, accuracy was 0.005 mmol/L (arginine) and 0.005 mg/L (glucose and xylose). For the sake of simplicity, significant figures relative to analyte concentrations are omitted from the text and mentioned only if strictly necessary. 

#### 3.2.1. Stock Solutions and Calibration Standards

Stock solutions (5 g/L) of glucose and xylose were prepared by dissolving each purchased standard in water in a 100-mL volumetric flask. To prepare calibration standards for HPAEC-IPAD, stock solutions were diluted in water to obtain concentrations ranging from 0.125 to 20 mg/L. A stock solution of arginine was prepared by diluting the purchased standard to 1 mmol/L. Calibration standards of 0.50–90 µmol/L (0.087–15.68 mg/L) were further prepared by diluting the stock solution in water. A stock solution containing 1 µmol/L of arginine was also prepared from the standard amino acid mixture. For RP-UHPLC-CAD, calibration standards and solutions for the linearity study were prepared by diluting glucose and xylose stock solutions to concentrations ranging from 1.56 mg/L to 2 g/L. Arginine solutions of 0.025–12 mmol/L (4.36 mg/L to 2.09 g/L) were prepared by diluting the purchased standard. Again, a stock solution containing 1 mmol/L of arginine was prepared from the amino acid standard. Stock and standard solutions were kept at 4 °C.

#### 3.2.2. Model Fermentation Samples

To assess suitability of the methods for fermentation sample analysis, model fermentation samples with known concentrations of glucose, xylose, and arginine or an amino acid mixture were analyzed. M9’ derived from the classical minimal M9 medium, LB, and CSL were used as media (Table 19). After sterilization at 120 °C for 20 min, media were diluted 1000 and 100 times for HPAEC-IPAD and RP-UHPLC-CAD, respectively, and filtered through 0.2-µm PP filters. The dilutions ensured that the concentrations of analytes in the real fermentation samples fell within the method range. Spiked media samples were prepared by aseptically diluting sterile-filtered stock solutions into the different media. 

#### 3.2.3. Fermentation Samples

To apply the methods to real fermentation samples, *E. coli* strain SJB009, previously engineered for an enhanced production of arginine, was used [13]. Two different fermentations were performed in 1-L bioreactors: one with M9’ and glucose (30 g/L) and another with CSL and xylose as substrate (50 g/L). The fermentations were carried out as detailed elsewhere [13,56]. Samples were taken at various times during the fermentations (beginning, middle, and end) to represent different ratios of arginine to substrate. The samples were centrifuged for 10 min at 4 °C and 10,600× *g*, and the supernatants were filtered through 0.2-µm PP filters. They were then diluted with water, typically 500 to 4000 times, to obtain arginine and sugar concentrations within the linearity range (0.5–20 µmol/L for arginine and 1–20 mg/L for the monosaccharides) for HPAEC-IPAD. For RP-UHPLC-CAD the samples were diluted to 25 µmol/L–7.5 mmol/L for arginine and 1.5 mg/L up to 1 g/L for glucose/xylose. 

Cultivation in LB was also monitored by inoculating a shake flask containing 100 mL sterile LB with 1 mL stock culture of *E. coli*, followed by incubation at 32 °C and 200 rpm. No additional substrate was provided. Samples were regularly taken following fermentation dynamics (e.g. 0, 3, 18, 21, 24 and 40 hours for the M9’ and glucose fermentation) and prepared in the same way as bioreactor samples. 

### 3.3. Instruments

Ion chromatography analyses were performed using the Dionex AAA-Direct^TM^ system (Thermo Scientific, San Jose, CA, USA) consisting of a Dionex-ICS 5000+ HPIC standard unit, with a Dionex™ ICS-5000+ SP gradient single pump, a Dionex™ AS-AP autosampler and a Dionex™ ICS-5000+ ED Electrochemical Detector. The electrochemical detector comprised of a pH, Ag/AgCl reference electrode, and a gold AAA-Direct disposable working electrode. Analytes were separated in an anion-exchange column (AminoPac^TM^PA10 Analytical Column, 4 × 250 mm,) preceded by a guard column (AminoPac^TM^PA10 Guard Column, 4 × 50 mm,) from Thermo Scientific (San Jose, CA, USA). 

Liquid chromatography analyses were performed using an UltiMate^TM^ 3000 rapid separation liquid chromatography (RSLC) system (Thermo Scientific, Germering, Germany) equipped with a dual-gradient standard pump UltiMate™ DGP-3600SD and a Corona Veo CAD. Analytes were separated on a reversed-phase column (Acclaim^TM^ RSLC Polar Advantage II, 2.2 µm and 2.1 × 250 mm, Thermo Scientific, San Jose, CA, USA). All data collection and processing was performed using Dionex *Chromeleon* 7.2.6 software (Thermo Scientific, Germering, Germany).

## 4. Conclusions

The high-performance anion-exchange chromatography combined with integrated pulse amperometric detection (HPAEC-IPAD) and reversed-phase liquid chromatography coupled to charged aerosol detection (RP-UHPLC-CAD) methods were fully validated for accurate, qualitative and quantitative determination of arginine, glucose, and/or xylose during microbial production of arginine from fermentable sugars. Excellent accuracy and precision in combination with an LOD of 0.1-2 µmol/L for arginine were obtained for both methods. 

For the first time this chromatographic approach was applied to study binary mixtures (arginine and monosaccharides) and further on genuine fermentation samples. Consequently, the arginine production during *E. coli* fermentation could be successfully monitored along with the sugar consumption. The approach was found highly suitable for arginine, glucose, and xylose determination in commonly used fermentation media, i.e., lysogeny broth (LB), minimal medium (M9’), and complex medium containing corn steep liquor (CSL).

The HPAEC-IPAD and RP-UHPLC-CAD methods do not require pre-column derivatization, while offering simultaneous and fast analysis of the main product and substrate(s). Hence, fermentation media rich in ammonia could be subjected to analysis using either the IPAD or CAD system.

Both HPAEC-IPAD and RP-UHPLC-CAD methods demonstrated high sensitivity that put forward the potential for amino acids to be independently quantified or in mixtures with ammonia and monosaccharides.

## Figures and Tables

**Table 1:** Instrument parameters for high-performance anion-exchange chromatography coupled to integrated pulsed amperometric detection (HPAEC-IPAD).

Time (min)	Water (%)	250 mM Sodium Hydroxide (%)	1 M Sodium Acetate (%)	Curve™ *
0.0	76	24	0	5
2.0	76	24	0	5
8.0	64	36	0	5
11.0	0	30	70	8
14.0	0	30	70	5
14.1	0	100	0	5
15.0	0	100	0	5
16.0	76	24	0	2
23.0	76	24	0	-

* Curve gradients were calculated by *Chromeleon* software (registered TM of Dionex).

**Table 2:** Instrument parameters for reversed-phase ultra-high-performance liquid chromatography combined with charged aerosol detection (RP-UHPLC-CAD).

Time (min)	Flow (mL/min)	0.4% HFBA (%)	Acetonitrile (%)	Curve™ *
−5.0	0.25	100	0	5
0.0	0.25	100	0	5
0.0 ^a^	0.25	100	0	5
3.0	0.35	100	0	5
4.5	0.45	100	0	5
10.0	0.45	75	25	5
12.0	0.45	75	25	5
13.0	0.45	55	45	7
14.0	0.45	55	45	5
16.0	0.25	100	0	4
17.0	0.25	100	0	-

^a^ 0.001 min due to software configuration. * Curve gradients were calculated by *Chromeleon* software (registered TM of Dionex). HFBA- Heptafluorobutyric acid.

**Table 3:** Evaluation of HPAEC-IPAD suitability for glucose, xylose, and arginine in M9’medium (*n* = 6).

Injection	Glucose (7.0 mg/L)		Xylose (7.0 mg/L)		Arginine (15.0 µmol/L)	
	ret. time (min)	area (pA × min)	ret. time (min)	area (pA × min)	ret. time (min)	area (pA × min)
#1	7.27	72.088	7.59	81.275	2.25	16.668
#2	7.24	72.990	7.56	82.239	2.25	16.076
#3	7.33	71.734	7.57	82.841	2.26	15.956
#4	7.28	72.170	7.58	82.554	2.26	16.030
#5	7.29	72.458	7.56	82.605	2.26	16.009
#6	7.37	72.186	7.58	83.084	2.25	15.997
Mean ± std	7.28 ± 0.05	72.271 ± 0.422	7.57 ± 0.01	82.433 ± 0.634	2.25 ± 0.01	16.122 ± 0.270
% RSD	0.63	0.58	0.16	0.77	0.24	1.67

**Table 4:** Evaluation of RP-UHPLC-CAD suitability for glucose, xylose, and arginine in M9’ medium (*n* = 6).

Injection	Glucose (0.100 g/L)		Xylose (0.100 g/L)		Arginine (0.75 mmol/L)	
	Ret. Time (min)	Area (pA × min)	Ret. Time (min)	Area (pA × min)	Ret. Time (min)	Area (pA × min)
#1	2.18	2.153	2.21	2.225	11.01	5.789
#2	2.18	2.124	2.23	2.216	11.01	5.757
#3	2.18	2.083	2.22	2.258	11.01	5.707
#4	2.18	2.104	2.22	2.223	11.01	5.748
#5	2.18	2.141	2.22	2.232	11.01	5.678
#6	2.18	2.091	2.22	2.214	11.01	5.863
Mean ± std	2.18 ± na	2.116 ± 0.026	2.22 ± 0.01	2.228 ± 0.015	11.01 ± na	5.757 ± 0.065
% RSD	0.08	1.21	0.26	0.66	0.02	1.13

na, not applicable: standard deviation (std) <0.01.

**Table 5:** Evaluation of HPAEC-IPAD specificity by comparing retention times in different media supplemented with glucose, xylose, or arginine (*n* = 3).

	Glucose		Xylose		Arginine	
Medium	Conc. (mg/L)	Ret. Time (min) Mean ± std	Conc. (mg/L)	Ret. Time (min) Mean ± std	Conc. (µmol/L)	Ret. Time (min) Mean ± std
H_2_O	2.0	7.28 ± 0.01	2.0	7.61 ± 0.02	10.0	2.26 ± na
	8.0	7.29 ± 0.02	8.0	7.62 ± 0.02	15.0	2.26 ± 0.01
	12.0	7.27 ± 0.02	12.0	7.61 ± 0.02	30.0	2.25 ± 0.01
	20.0	7.21 ± 0.02	20.0	7.56 ± 0.01	45.0	2.26 ± 0.01
	Mean ± std	7.27 ± 0.04	Mean ± std	7.60 ± 0.02	Mean ± std	2.26 ± 0.01
	%RSD	0.49	%RSD	0.28	%RSD	0.12
M9’	2.5	7.25 ± 0.01	2.5	7.67 ± 0.08	5.0	2.260 ± na
	7.0	7.28 ± 0.05	7.0	7.57 ± 0.01	15.0	2.25 ± 0.01
	12.0	7.24 ± 0.01	12.0	7.52 ± 0.02	15.0 ^a^	2.26 ± 0.01
					30	2.24 ± 0.01
	Mean ± std	7.25 ± 0.02	Mean ± std	7.59 ± 0.08	Mean ± std	2.25 ± 0.01
	%RSD	0.31	%RSD	1.03	%RSD	0.32
LB	2.5	7.22 ± 0.01	2.5	7.63 ± 0.07	5.0	2.26 ± 0.01
	7.0	7.26 ± 0.04	7.0	7.56 ± 0.01	15.0	2.25 ± na
	12.0	7.21 ± 0.05	12.0	7.63 ± 0.07	15.0 ^a^	2.25 ± 0.01
					30	2.24 ± 0.01
	Mean ± std	7.23 ± 0.03	Mean ± std	7.61 ± 0.04	Mean ± std	2.25 ± 0.01
	%RSD	0.37	%RSD	0.52	%RSD	0.25
CSL	2.5	7.23 ± 0.02	2.5	7.64 ± 0.08	5.0	2.25 ± na
	7.0	7.29 ± 0.03	7.0	7.57 ± 0.02	15.0	2.25 ± 0.01
	12.0	7.27 ± 0.04	12.0	7.55 ± 0.01	15.0 ^a^	2.25 ± 0.01
					30.0	2.25 ± na
	Mean ± std	7.27 ± 0.03	Mean ± std	7.58 ± 0.05	Mean ± std	2.25 ± 0.01
	%RSD	0.44	%RSD	0.61	%RSD	0.12
Overall ^b^	Mean ± std	7.26 ± 0.03	Mean ± std	7.59 ± 0.04	Mean ± std	2.25 ± 0.01
	%RSD	0.43	%RSD	0.55	%RSD	0.25

na, not applicable: standard deviation (std) <0.01. ^a^ arginine from an amino acid standard mixture test. ^b^ verall data: calculated based on the mean for each medium.

**Table 6:** Evaluation of RP-UHPLC-CAD specificity by comparing retention times in different media supplemented with glucose, xylose or arginine (*n* = 3).

	Glucose		Xylose		Arginine	
Medium	Conc. (g/L)	Ret. Time (min) Mean ± std	Conc.(g/L)	Ret. Time (min) Mean ± std	Conc. (mmol/L)	Ret. Time (min) Mean ± std
H_2_O	0.0125	2.17 ± na	0.0125	2.22 ± na	0.025	11.05 ± 0.01
	0.10	2.18 ± na	0.10	2.22 ± 0.01	0.10	11.05 ± na
	1.00	2.18 ± na	1.00	2.22 ± 0.01	0.75	11.02 ± na
	2.00	2.18 ± na	2.00	2.22 ± na	7.50	10.87 ± 0.01
	Mean ± std	2.18 ± na	Mean ± std	2.22 ± na	Mean ± std	11.00 ± 0.09
	%RSD	0.07	%RSD	0.14	%RSD	0.77
M9’	0.01	2.17 ± 0.02	0.01	2.21 ± 0.01	0.125	11.04 ± 0.02
	0.10	2.18 ± na	0.10	2.22 ± 0.01	0.75	11.01 ± na
	1.00	2.18 ± 0.01	1.00	2.22 ± 0.01	0.750 ^a^	11.00 ± na
					7.50	10.86 ± 0.01
	Mean ± std	2.18 ± 0.01	Mean ± std	2.22 ± 0.01	Mean ± std	10.98 ± 0.06
	%RSD	0.31	%RSD	0.19	%RSD	0.73
LB	0.01	2.17 ± na	0.01	2.22 ± na	0.125	11.03 ± na
	0.10	2.18 ± na	0.10	2.22 ± na	0.75	11.01 ± 0.01
	1.00	2.18 ± na	1.00	2.22 ± 0.01	0.750 ^a^	11.02 ± 0.02
					7.50	10.85 ± 0.01
	Mean ± std	2.18 ± na	Mean ± std	2.22 ± na	Mean ± std	10.97 ± 0.08
	%RSD	0.11	%RSD	0.16	%RSD	0.77
CSL	0.01	2.18 ± na	0.01	2.21 ± na	0.125	11.03 ± 0.01
	0.10	2.19 ± na	0.10	2.22 ± 0.01	0.75	11.01 ± na
	1.00	2.18 ± na	1.00	2.22 ± na	0.750 ^a^	11.01 ± na
					7.50	10.85 ± 0.02
	Mean ± std	2.18 ± na	Mean ± std	2.22 ± na	Mean ± std	10.98 ± 0.09
	%RSD	0.17	%RSD	0.15	%RSD	0.76
Overall ^b^	Mean ± std	2.18 ± na	Mean ± std	2.22 ± na	Mean ± std	10.98 ± 0.01
	%RSD	0.11	%RSD	0.09	%RSD	0.09

na, not applicable: standard deviation (std) <0.01. ^a^ arginine from a standard amino acids mixture. ^b^ overall data: calculated based on the mean for each medium.

**Table 7:** Calibration curve details including limit of quantification (LOQ) and limit of detection (LOD) for HPAEC-IPAD.

	Evaluation Parameter	Calibration Type	Range	Intercept	Slope	Curvature ^a^ ™	*R^2^*	LOQ	LOD
Arginine	Area	Quadratic with Offset	1.5–30 µmol/L	0.085	1.225	−0.0092	0.9973	0.3 µmol/L	0.1 µmol/L
Glucose	Area	Linear	2–20 mg/L	1.586	10.193	0	0.9991	1 mg/L	30 µg/L
Xylose	Area	Linear	1–20 mg/L	−0.085	11.514	0	0.9997	0.25 mg/L	8 µg/L

^a^ Curve fitting was calculated by the method of least squares using *Chromeleon* software (TM of Dionex).

**Table 8:** Calibration curves details with limit of quantification (LOQ) and limit of detection (LOD) for RP-UHPLC-CAD.

	Evaluation Type	Calibration Type	Range	Intercept	Curvature ^a^	Slope	*R^2^*	LOQ	LOD
Arginine	Area	Quadratic with Offset	25 µmol/L–7.5 mmol/L	−0.066	0.137	0.0028	0.9998	10 µmol/L	2 µmol/L
Glucose	Area	Quadratic with Offset	1.5 mg/L–1 g/L	−0.002	0.037	0.0113	0.9993	1 mg/L	0.3 mg/L
Xylose	Area	Quadratic with Offset	1.5 mg/L–1 g/L	−0.004	0.044	0.0069	0.9994	1 mg/L	0.4 mg/L

^a^ Curve fitting was calculated by the method of least squares using *Chromeleon* software (TM of Dionex).

**Table 9:** Arginine recovery in different media (*n* = 3) for HPAEC-IPAD.

	Arginine		
	M9’	LB	CSL
5.0 µmol/L			
Inj #1	4.85	7.16	12.77
Inj #2	4.92	6.81	12.39
Inj #3	4.97	7.04	12.26
Mean ± std	4.91 ± 0.06	7.00 ± 0.17	12.47 ± 0.26
% RSD	1.16	2.49	2.11
% Recovery	98.28	102.67 ^b^	249.48
15.0 µmol/L			
Inj #1	15.28	16.64	22.83
Inj #2	15.30	16.36	22.28
Inj #3	14.56	17.09	22.84
Mean ± std	15.05 ± 0.42	16.70 ± 0.37	22.65 ± 0.32
% RSD	2.80	2.21	1.41
% Recovery	100.33	99.28 ^b^	150.99
15.00 ^a^ µmol/L			
Inj #1	15.01	16.52	22.63
Inj #2	15.69	15.61	22.32
Inj #3	15.01	16.43	22.68
Mean ± std	15.24 ± 0.39	16.19 ± 0.50	22.55 ± 0.20
% RSD	2.56	3.10	0.87
% Recovery	101.58	96.23 ^b^	150.30
30.0 µmol/L			
Inj #1	29.50	32.08	38.91
Inj #2	28.79	31.84	37.33
Inj #3	28.52	30.98	37.08
Mean ± std	28.94 ± 0.51	31.63 ± 0.58	37.78 ± 0.99
% RSD	1.76	1.83	2.63
% Recovery	96.46	99.41 ^b^	125.92

^a^ Arginine from a standard amino acid mixture. ^b^ 1.82 µmol/L of arginine were added to the expected value (see Section 2.2.3); 1000 times dilution accounted for.

**Table 10:** Glucose and xylose recovery in different media (*n* = 3) for HPAEC-IPAD.

	Glucose			Xylose		
	M9’	LB	CSL	M9’	LB	CSL
2.5 mg/L						
Inj #1	2.51	2.52	2.44	2.65	2.52	2.59
Inj #2	2.59	2.60	2.65	2.60	2.49	2.54
Inj #3	2.48	2.57	2.45	2.58	2.59	2.58
Mean ± std	2.52 ± 0.06	2.56 ± 0.04	2.51 ± 0.12	2.61 ± 0.04	2.53 ± 0.05	2.57 ± 0.03
%RSD	2.30	1.64	4.83	1.43	2.14	1.17
%Recovery	100.98	102.57	100.55	104.35	101.26	102.80
7 mg/L						
Inj #1	6.99	7.05	7.01	7.06	7.36	6.93
Inj #2	6.93	7.13	7.04	7.15	7.08	6.94
Inj #3	6.91	6.95	6.99	7.17	7.34	6.87
Mean ± std	6.94 ± 0.04	7.05 ± 0.09	7.01 ± 0.03	7.13 ± 0.06	7.26 ± 0.16	6.91 ± 0.04
%RSD	0.57	1.28	0.36	0.81	2.19	0.58
%Recovery	99.18	101.13	100.19	101.84	103.71	98.76
12 mg/L						
Inj #1	12.01	12.19	12.02	12.00	12.44	12.31
Inj #2	11.98	11.71	11.88	12.31	12.02	12.32
Inj #3	11.98	11.74	12.04	12.15	12.50	12.25
Mean ± std	11.99 ± 0.01	11.88 ± 0.27	11.98 ± 0.09	12.15 ± 0.16	12.32 ± 0.26	12.29 ± 0.04
%RSD	0.12	2.26	0.75	1.28	2.10	0.33
%Recovery	99.92	98.98	99.83	101.29	102.65	102.45

**Table 11:** Arginine recovery in different media (*n* = 3) for RP-UHPLC-CAD.

	Arginine (mmol/L)		
	M9’	LB	CSL
Expected: 0.125			
Inj #1	0.124	0.148	0.128
Inj #2	0.129	0.149	0.132
Inj #3	0.131	0.149	0.132
Mean ± std	0.128 ± 0.004	0.149 ± 0.001	0.131 ± 0.002
%RSD	3.07	0.60	1.48
%Recovery	102.40	103.79 ^b^	104.51
Expected: 0.750			
Inj #1	0.808	0.828	0.792
Inj #2	0.815	0.821	0.790
Inj #3	0.803	0.846	0.780
Mean ± std	0.809 ± 0.006	0.832 ± 0.013	0.788 ± 0.006
%RSD	0.73	1.52	0.78
%Recovery	107.80	108.26 ^b^	104.99
Expected: 0.750 ^a^			
Inj #1	0.803	0.813	0.818
Inj #2	0.781	0.791	0.808
Inj #3	0.806	0.817	0.810
Mean ± std	0.797 ± 0.013	0.807 ± 0.014	0.812 ± 0.006
%RSD	1.66	1.73	0.69
%Recovery	106.22	105.03 ^b^	108.27
Expected: 7.50			
Inj #1	7.668	7.601	7.648
Inj #2	7.803	7.636	7.577
Inj #3	7.795	7.592	7.962
Mean ± std	7.755 ± 0.076	7.610 ± 0.023	7.729 ± 0.205
%RSD	0.97	0.31	2.65
%Recovery	103.40	101.21 ^b^	103.05

^a^ Arginine from a standard amino acid mixture. ^b^ 0.0182 mmol/L of arginine were added to the expected value in 100 times diluted medium.

**Table 12:** Glucose and xylose recovery in different media (*n* = 3) for RP-UHPLC-CAD.

	Glucose (g/L)			Xylose (g/L)		
	M9’	LB	CSL	M9’	LB	CSL
Expected	0.01	0.05	0.05	0.01	0.01	0.05
Inj #1	0.01	0.05	0.05	0.01	0.01	0.05
Inj #2	0.01	0.05	0.05	0.01	0.01	0.05
Inj #3	0.01	0.05	0.05	0.010	0.01	0.05
Mean ± std	0.01 ± na	0.05 ± na	0.052 ± na	0.01 ± na	0.01 ± na	0.054 ± na
%RSD	3.25	0.33	0.63	3.49	1.71	0.70
%Recovery	101.33	98.60	104.13	105.33	99.67	108.07
Expected	0.10	0.10	0.10	0.10	0.10	0.10
Inj #1	0.11	0.11	0.11	0.10	0.11	0.11
Inj #2	0.11	0.11	0.11	0.10	0.10	0.11
Inj #3	0.11	0.11	0.11	0.10	0.11	0.11
Mean ± std	0.11 ± na	0.11 ± na	0.11 ± na	0.10 ± na	0.11 ± na	0.11 ± na
%RSD	0.58	0.31	1.12	0.24	1.08	0.72
%Recovery	106.37	108.17	107.40	103.57	105.57	109.47
Expected	1.00	1.00	1.00	1.00	1.00	1.00
Inj #1	1.12	1.01	1.04	0.95	0.98	1.01
Inj #2	1.04	1.05	1.10	0.98	0.98	1.00
Inj #3	1.03	1.05	1.07	1.01	1.01	0.98
Mean ± std	1.06 ± 0.04	1.04 ± 0.02	1.07 ± 0.03	0.98 ± 0.03	0.99 ± 0.02	0.999 ± 0.01
%RSD	3.68	1.72	2.51	2.52	1.52	1.17
%Recovery	106.35	103.69	106.94	97.91	99.11	99.89

na, not applicable: standard deviation (std) <0.01.

**Table 13:** Intermediate precision for HPAEC-IPAD in CSL medium (*n* = 3).

	Day 1	Day 2	Day 3	Mean ± std	%RSD
Arginine (15.0 µmol/L)	20.21 ± 0.35	19.53 ± 0.10	19.66 ± 0.33	19.80 ± 0.36	1.84
Glucose (7.0 mg/L)	6.93 ± 0.01	7.01 ± 0.03	7.07 ± 0.13	7.00 ± 0.07	1.05
Xylose (7.0 mg/L)	6.68 ± 0.11	6.91 ± 0.04	6.55 ± 0.12	6.72 ± 0.18	2.73

**Table 14:** Intermediate precision for RP-UHPLC-CAD evaluated in CSL medium (*n* = 3).

	Day 1	Day 2	Day 3	Mean ± std	%RSD
Arginine (0.75 mmol/L)	0.787 ± 0.006	0.810 ± 0.013	0.793 ± 0.001	0.797 ± 0.012	1.48
Glucose (0.100 g/L)	0.105 ± 0.001	0.106 ± 0.001	0.102 ± 0.002	0.104 ± 0.002	1.61
Xylose (0.100 g/L)	0.109 ± 0.001	0.109 ± 0.001	0.110 ± 0.001	0.109 ± 0.001	0.46

**Table 15:** Recovery of arginine/glucose and arginine/xylose in different media (*n* = 2) with HPAEC-IPAD.

Medium	Arginine (µmol/L)			Glucose (mg/L)		
	Expected	Mean ± std	%Recovery	Expected	Mean ± std	%Recovery
H_2_O	5.0	4.87 ± 0.01	97.38	20.0	19.74 ± 0.19	98.68
H_2_O	15.0	14.44 ± 0.10	96.29	10.0	10.28 ± 0.31	102.80
H_2_O ^a^	15.0	14.89 ± na	99.24	10.0	10.40 ± 0.04	104.00
M9’	5.0	4.97 ± 0.01	99.34	20.0	20.02 ± 0.01	100.08
M9’	15.0	14.79 ± 0.08	98.60	10.0	9.61 ± 0.08	96.12
M9’ ^a^	15.0	14.61 ± 0.24	97.38	10.0	10.19 ± 0.19	101.91
LB	6.82 ^b^	6.72 ± 0.30	98.52	20.0	19.90 ± 0.01	99.48
LB	16.82 ^b^	16.55 ± 0.56	98.40	10.0	10.45 ± 0.29	104.53
LB ^a^	16.82 ^b^	16.99 ± 0.12	100.99	10.0	10.24 ± 0.06	102.36
**Media**	**Arginine (µmol/L)**			**Xylose (mg/L)**		
	**Expected**	**Mean ± std**	**%Recovery**	**Expected**	**Mean ± std**	**%Recovery**
H_2_O	5.0	4.98 ± 0.09	99.60	20.0	19.45 ± 0.17	97.25
H_2_O	15.0	15.24 ± 0.04	101.61	10.0	10.22 ± 0.04	102.17
H_2_O ^a^	15.0	15.01 ± 0.34	100.05	10.0	9.75 ± 0.13	97.47
M9’	5.0	4.96 ± 0.13	99.28	20.0	19.56 ± 0.52	97.78
M9’	15.0	14.84 ± 0.05	98.91	10.0	9.99 ± 0.13	99.88
M9’ ^a^	15.0	14.79 ± 0.12	98.63	10.0	9.63 ± 0.03	96.31
LB	6.8 ^b^	6.74 ± 0.02	98.84	20.0	18.87 ± 0.53	94.37
LB	16.8 ^b^	16.75 ± 0.15	99.59	10.0	10.02 ± 0.15	100.12
LB ^a^	16.8 ^b^	16.80 ± 0.08	99.88	10.0	9.87 ± 0.12	98.71

na, not applicable: standard deviation (std) <0.01. ^a^ arginine from a standard amino acid mixture. ^b^ expected value was corrected with an initial 1.82 µmol/L arginine for 1000 times diluted medium.

**Table 16:** Recovery of ternary mixtures using HPAEC-IPAD.

Medium	Arginine (µmol/L)			Glucose (mg/L)			Xylose (mg/L)		
	Exp.	Mean ± Std	%Recovery	Exp.	Mean ± Std	%Recovery	Exp.	Mean ± Std	%Recovery
H_2_O	15.0	15.07 ± 0.18	100.49	10.0	9.94 ± 0.90	99.37	10.0	10.31 ± 0.72	103.07
M9’	15.0	14.92 ± 0.31	99.49	10.0	9.74 ± 0.79	100.86	10.0	10.09 ± 0.70	100.86
LB	16.8 ^a^	16.50 ± 0.35	98.11	10.0	9.53 ± 0.40	95.27	10.0	9.56 ± 0.39	95.58

^a^ Expected value was corrected with an initial 1.82 µmol/L arginine for 1000 times diluted medium.

**Table 17:** Recovery of arginine/glucose and arginine/xylose in different media (*n* = 3) with RP-UHPLC-CAD.

Medium	Arginine (mmol/L)			Glucose (g/L)		
	Expected	Mean ± std	%Recovery	Expected	Mean ± std	%Recovery
H_2_O	0.125	0.128 ± na	102.40	1.00	1.00 ± 0.01	99.97
	0.75	0.80 ± 0.02	107.08	0.10	0.10 ± na	99.73
	7.50	6.77 ± 0.06	90.23	0.01	0.01 ± na	93.67
M9’	0.125	0.127 ± na	101.57	1.00	1.09 ± 0.02	108.90
	0.75	0.78 ± na	104.92	0.10	0.09 ± na	97.63
	7.50	7.65 ± 0.06	101.99	0.01	0.01 ± na	101.00
LB	0.143 ^a^	0.145 ± na	101.58	1.00	1.06 ± 0.01	106.42
	0.768 ^a^	0.82 ± na	106.27	0.10	0.11 ± na	106.30
	7.518 ^a^	7.62 ± 0.02	101.34	0.05	0.05 ± na	96.80
CSL	0.125	0.116 ± na	92.69	1.00	1.04 ± 0.05	103.70
	0.75	0.78 ± 0.02	104.93	0.10	0.11 ± na	107.67
	7.50	7.67 ± 0.01	102.31	0.05	0.05 ± na	102.07
**Medium**	**Arginine (mmol/L)**			**Xylose (g/L)**		
	**Expected**	**Mean ± std**	**%Recovery**	**Expected**	**Mean ± std**	**%Recovery**
H_2_O	0.125	0.124 ± na	98.91	1.00	0.98 ± na	98.08
	0.75	0.73 ± 0.01	97.68	0.10	0.09 ± na	94.20
	7.50	6.99 ± 0.01	93.27	0.01	0.01 ± na	97.00
M9’	0.125	0.115 ± na	92.03	1.00	0.96 ± na	96.54
	0.75	0.74 ± na	98.97	0.10	0.09 ± na	96.10
	7.50	7.45 ± 0.09	99.30	0.01	0.01 ± na	105.67
LB	0.143 ^a^	0.133 ± 0.01	92.90	1.00	0.97 ± 0.02	97.65
	0.768 ^a^	0.81 ± 0.01	105.28	0.10	0.11 ± na	105.00
	7.518 ^a^	7.58 ± 0.11	100.81	0.05	0.05 ± na	105.00
CSL	0.125	0.123 ± na	98.67	1.00	1.01 ± 0.02	101.35
	0.75	0.69 ± 0.01	92.31	0.10	0.10 ± na	101.90
	7.50	7.52 ± 0.04	100.25	0.05	0.05 ± na	109.27

na, not applicable: standard deviation (std) <0.01. ^a^ expected value was corrected with an initial 0.0182 mmol/L arginine for 100 times diluted medium.

**Table 18:** Comparison of the HPAEC-IPAD and RP-UHPLC-CAD systems for arginine and carbohydrates analysis in pure water and in fermentation samples.

	RP-UHPLC-CAD	HPAEC-IPAD
Method		
Eluents	0.4% HFBA, acetonitrile	water, 250 mM NaOH, 1 M NaOAc
Flow rate	0.25–0.45 mL/min	0.75 mL/min
Injection volume	5 µL	25 µL
Total run time	22 min	23 min
Start-up routine	15 min with only nitrogen generator on. Pump priming. Gradual increase of flow rate up to initial method value. 5 min equilibration check for pump pressure and detector signal.	Eluents filtered through 0.45-µM filters and degassed 5 min with argon. Disposable gold electrode (change every week). Ref. electrode pH calibration. Pump priming. Gradual increase of flow rate up to method value. 5 min equilibration check for pump pressure and detector signal.
Analysis (pure water)		
Arginine		
Retention time	10.98 min	2.26 min
Range	2–1300 mg/L (20–6500 ng on column)	0.26–5.2 mg/L (6.5–131 ng on column)
LOQ	1.74 mg/L (8.7 ng on column)	52 µg/L (1.3 ng on column)
LOD	0.35 mg/L (1.7 ng on column)	17 µg/L (0.42 ng on column)
Glucose		
Retention time	2.17 min	7.27 min
Range	1.5–1000 mg/L (7.5–5000 ng on column)	2–20 mg/L (50–500 ng on column)
LOQ	1 mg/L (5 ng on column)	1 mg/L (25 ng on column)
LOD	0.3 mg/L (1.5 ng on column)	30 µg/L (0.75 ng on column)
Xylose		
Retention time	2.22 min	7.60 min
Range	1.5–1000 mg/L (7.5–5000 ng on column)	1–20 mg/L (25–500 ng on column)
LOQ	1 mg/L (5 ng on column)	0.25 mg/L (6.3 ng on column)
LOD	0.4 mg/L (2.5 ng on column)	8 µg/L (0.2 ng on column)
Performance (fermentation media)		
Arginine		
Accuracy ^a^	101.2–104.3%	96.2–102.7%
Repeatability ^b^	0.3–3.1%	1.2–3.1%
Intermediate precision ^c^	1.5%	1.8%
Media	M9’, LB, CSL	M9’, LB
Glucose		
Accuracy ^a^	91.5–108.3%	99.0–102.6%
Repeatability ^b^	1.1–4.4%	0.1–4.8%
Intermediate precision ^c^	2.5%	1.1%
Media	M9’, LB ^d^, CSL ^d^	M9’, LB, CSL
Xylose		
Accuracy ^a^	96.2–105.8%	98.8–104.3%
Repeatability ^b^	0.2–3.5%	0.3–2.2%
Intermediate precision ^c^	1.0%	2.7%
Media	M9’, LB, CSL ^d^	M9’, LB, CSL

^a^ Accuracy expressed as percentage recovery range. ^b^ repeatability expressed as %RSD range between triplicate samples. ^c^ intermediate precision expressed as %RSD between three series of triplicates analyzed on three different days. ^d^ lowest concentration with suitable accuracy = 0.05 g/L due to the presence of interfering components.

**Table 19:** Media composition.

Component	CSL (per L)	M9’ (per L)	LB (per L)
Corn steep liquor	15 g	-	-
(NH_4_)_2_SO_4_	15 g	5 g	-
KH_2_PO_4_	1 g	3 g	-
Na_2_HPO_4_	-	12.8 g	-
NaCl	-	0.5 g	10 g
MgSO_4_·7H_2_O	0.5 g	1 g ^a^	-
FeSO_4_·7H_2_O	20 mg	20 mg ^a^	-
MnSO_4_·H_2_O	12 mg	12 mg ^a^	-
CaCl_2_	-	1 mg ^a^	-
Antifoam	0.4 g	0.2 g	-
Tryptone	-	-	10 g
Yeast extract	-	-	5 g
Thiamine·HCl	0.5 mg ^a^	0.5 mg ^a^	-
Tetracycline	20 mg ^a^	20 mg ^a^	20 mg ^a^

^a^ Added after sterilization.

## Supplementary Materials

The following are available online. Table S1: Evaluation of HPAEC-IPAD specificity based on comparing retention times in water (*n* = 3), Table S2: Evaluation of RP-UHPLC-CAD specificity based on comparing retention times in water (*n* = 3), Figure S1: Calibration curve for glucose, and the effect of time and electrodes on the response using HPAEC-IPAD, Figure S2: Calibration curve for xylose, and the effect of time and electrodes on the response using HPAEC-IPAD, Figure S3: Calibration curve for arginine using HPAEC-IPAD, Figure S4: Calibration curve for arginine using RP-UHPLC-CAD, Figure S5: Calibration curve for glucose and the effect of time on the response using RP-UHPLC-CAD, Figure S6: Calibration curve for xylose and effect of time on the response using RP-UHPLC-CAD, Figure S7: Fermentation profile: cell growth, xylose consumption, and arginine production in CSL. Optical density (blue crosses), xylose (red squares), and arginine (green circles) were determined by RP-UHPLC-CAD (solid lines) or HPAEC-IPAD (dashed lines). Standard deviations are omitted for clarity.
