# Supplementary material for: Simultaneous Quantification of L-Arginine and Monosaccharides during Fermentation: An Advanced Chromatography Approach
Source: Molecules. 2019 Feb 22;24(4):802. doi: 10.3390/molecules24040802 (PMC6413082; doi:10.3390/molecules24040802)
Supplement: Supplementary file 1 [file molecules-24-00802-s001.pdf]

Supplementary Materials

Simultaneous Quantification of L-arginine and Monosaccharides during Fermentation: An Advanced Chromatography Approach

Mireille Ginésy [1], Josefine Enman [1,*], Daniela Rusanova-Naydenova [1], and Ulrika Rova [1]

[1] Biochemical Process Engineering, Division of Chemical Engineering, Department of Civil, Environmental and Natural Resources Engineering, Luleå University of Technology, SE-971 87 Luleå, Sweden; mireille.ginesy@gmail.com, josefine.enman@ltu.se, droussanova@hotmail.com, ulrika.rova@ltu.se

* Correspondence: josefine.enman@ltu.se; Tel +46- 920-49 12 67

Table 1. Evaluation of HPAEC-IPAD specificity based on comparing retention times in water (*n* = 3).

Glucose		Xylose		Arginine	
**Conc. (mg/L)**	**Ret. time (min) Mean ± Std**	**Conc. (mg/L)**	**Ret. time (min) Mean ± Std**	**Conc. (μmol/L)**	**Ret. time (min) Mean ± Std**
0.125	7.23 ± 0.02	0.125	7.50 ± 0.02	0.50	2.26 ± 0.01
0.25	7.28 ± 0.02	0.25	7.62 ± 0.04	0.75	2.26 ± na
0.50	7.28 ± 0.02	0.50	7.62 ± 0.02	1.0	2.26 ± 0.01
1.00	7.29 ± 0.02	1.00	7.61 ± 0.02	1.5	2.26 ± na
2.00	7.28 ± 0.01	2.00	7.61 ± 0.02	3.0	2.26 ± na
4.00	7.29 ± 0.03	4.00	7.61 ± 0.02	5.0	2.26 ± na
6.00	7.29 ± 0.02	6.00	7.61 ± na	7.5	2.26 ± 0.01
8.00	7.29 ± 0.02	8.00	7.62 ± 0.02	10	2.26 ± na
10.00	7.28 ± 0.03	10.00	7.61 ± 0.02	15	2.26 ± 0.01
12.00	7.27 ± 0.02	12.00	7.61 ± 0.02	30	2.25 ± 0.01
14.00	7.27 ± 0.02	14.00	7.58 ± 0.03	45	2.26 ± 0.01
16.00	7.28 ± 0.03	16.00	7.58 ± 0.02	60	2.26 ± 0.01
18.00	7.27 ± 0.02	18.00	7.57 ± 0.01	75	2.28 ± 0.01
20.00	7.21 ± 0.02	20.00	7.56 ± 0.01	90	2.29 ± 0.01
Mean ± Std	7.27 ± 0.02	Mean ± Std	7.60 ± 0.03	Mean ± Std	2.26 ± 0.03
%RSD	0.34	%RSD	0.41	%RSD	0.21

na, not applicable: standard deviation (std) < 0.01.

Table S1. Evaluation of RP-UHPLC-CAD specificity based on comparing retention times in water (*n* = 3).

Glucose		Xylose		Arginine	
**Conc. (mg/L)**	**Ret. time (min) Mean ± std**	**Conc. (mg/L)**	**Ret. time (min) Mean ± std**	**Conc. (μmol/L)**	**Ret. time (min) Mean ± std**
1.5625×10^{-3}	2.17 ± na	1.5625×10^{-3}	2.21 ± na	0.025	11.05 ± 0.01
3.125×10^{-3}	2.17 ± na	3.125×10^{-3}	2.21 ± na	0.05	11.06 ± 0.01
6.25×10^{-3}	2.17 ± na	6.25×10^{-3}	2.21 ± 0.01	0.075	11.06 ± 0.01
0.0125	2.17 ± na	0.0125	2.22 ± na	0.10	11.05 ± na
0.025	2.17 ± na	0.025	2.21 ± na	0.25	11.04 ± na
0.05	2.17 ± na	0.05	2.22 ± 0.01	0.50	11.03 ± na
0.075	2.18 ± na	0.075	2.22 ± 0.01	0.75	11.02 ± na
0.1	2.18 ± na	0.1	2.22 ± 0.01	1.0	11.01 ± 0.01
0.2	2.17 ± na	0.2	2.21 ± na	2.5	10.97 ± 0.01
0.4	2.18 ± na	0.4	2.22 ± na	5.0	10.91 ± na

0.6	2.18 ± na	0.6	2.22 ± na	7.5	10.87 ± 0.01
0.8	2.17 ± na	0.8	2.21 ± 0.01	10.0	10.84 ± na
1.0	2.17 ± na	1.0	2.22 ± 0.01	12.5	10.80 ± na
Mean ± std	2.17 ± na	Mean ± std	2.22 ± na	Mean ± std	10.97 ± 0.09
%RSD	0.14	%RSD	0.15	%RSD	0.84

na, not applicable: standard deviation (std) < 0.01

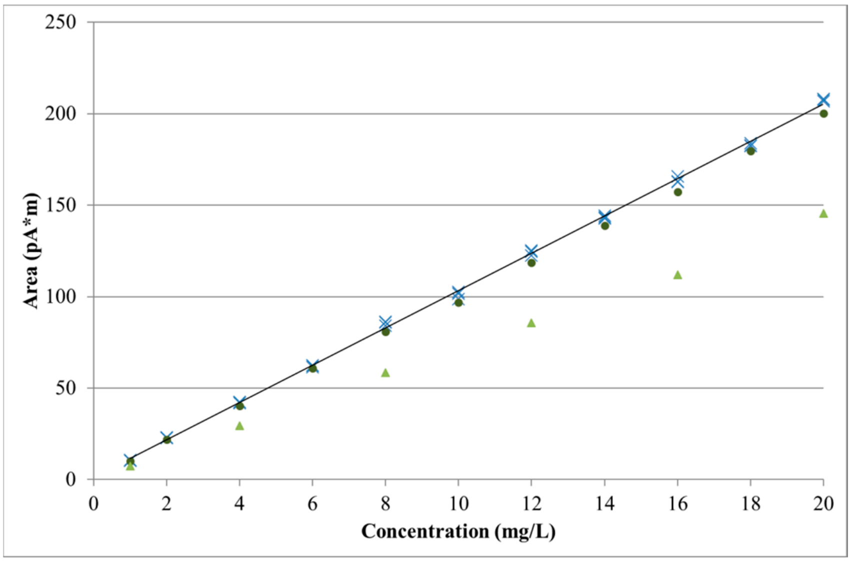

Figure S1. Calibration curve for glucose, and the effect of time and electrodes on the response using HPAEC-IPAD. Triplicates of standards analyzed within 24 h using the same new electrode (blue crosses) with the corresponding linear fitting curve (solid line); standards analyzed with another electrode: new (dark green circles) and after 43 h (green triangles) of continuous use.

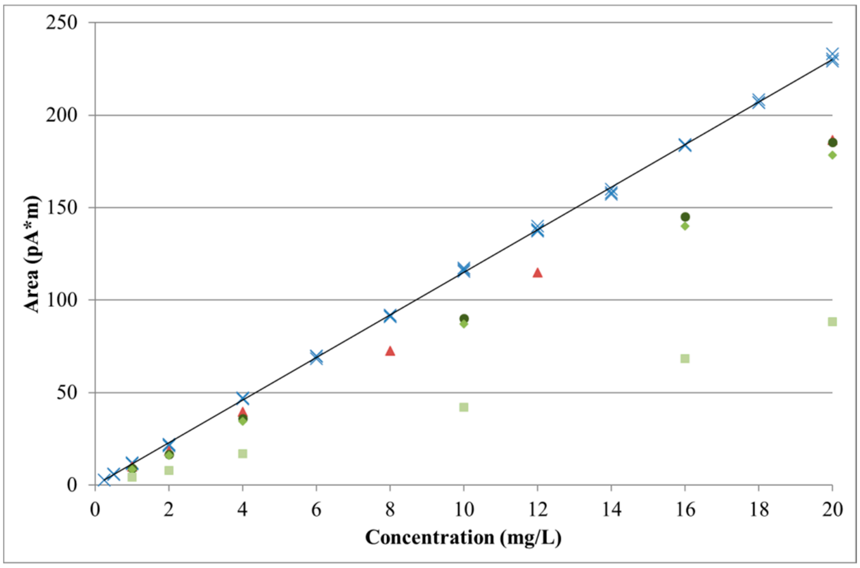

Figure S2. Calibration curve for xylose, and the effect of time and electrodes on the response using HPAEC-IPAD. Triplicates of standards analyzed within 24 h using the same new electrode (blue crosses) with the corresponding linear fitting curve (solid line); standards analyzed with another new electrode (red triangles); standards analyzed with a third electrode: new (dark green circles), after 14 h (green tilted squares), and after 35 h (light green squares) of continuous use.

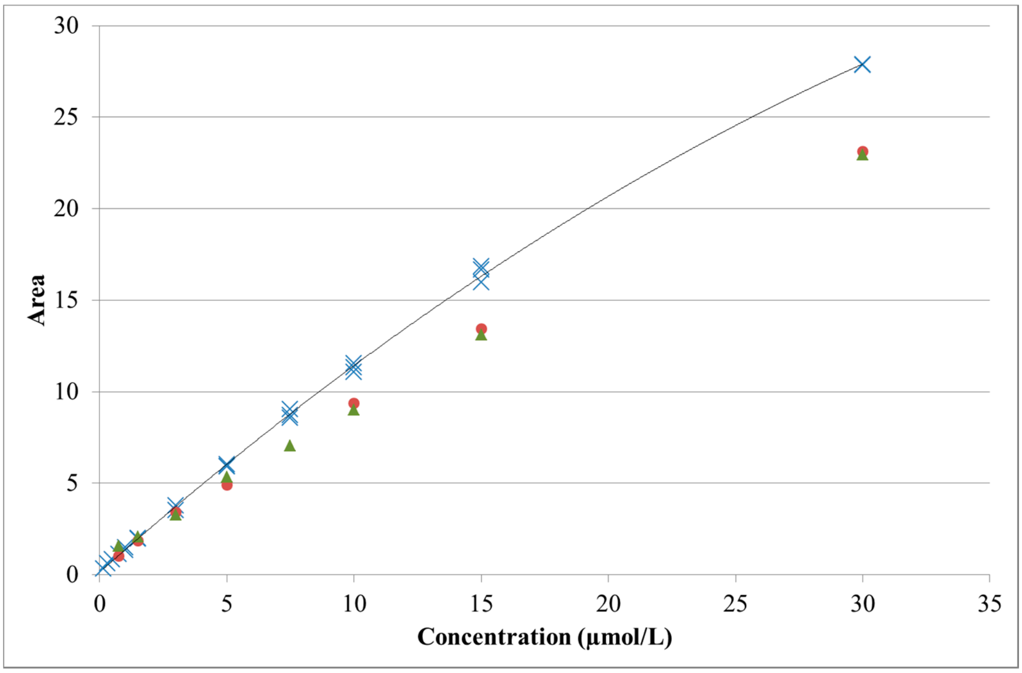

Figure S3. Calibration curve for arginine using HPAEC-IPAD. Triplicates of standards analyzed within 24 h using the same new electrode (blue crosses) with the corresponding quadratic fitting curve (solid line); standards analyzed with another new electrode (red circles); standards analyzed with a third new electrode (green triangles).

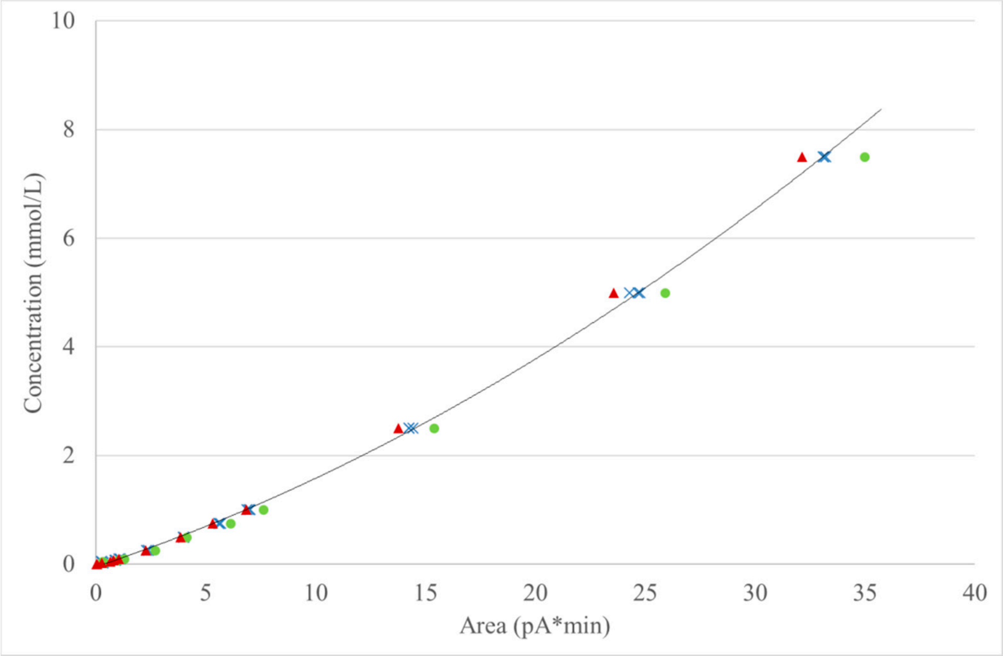

Figure S4. Calibration curve for arginine using RP-UHPLC-CAD. Triplicate standards analyzed within 24 h (blue crosses) with the corresponding quadratic fitting curve (solid line); standards analyzed one month later (green circles) and five months later (red triangles).

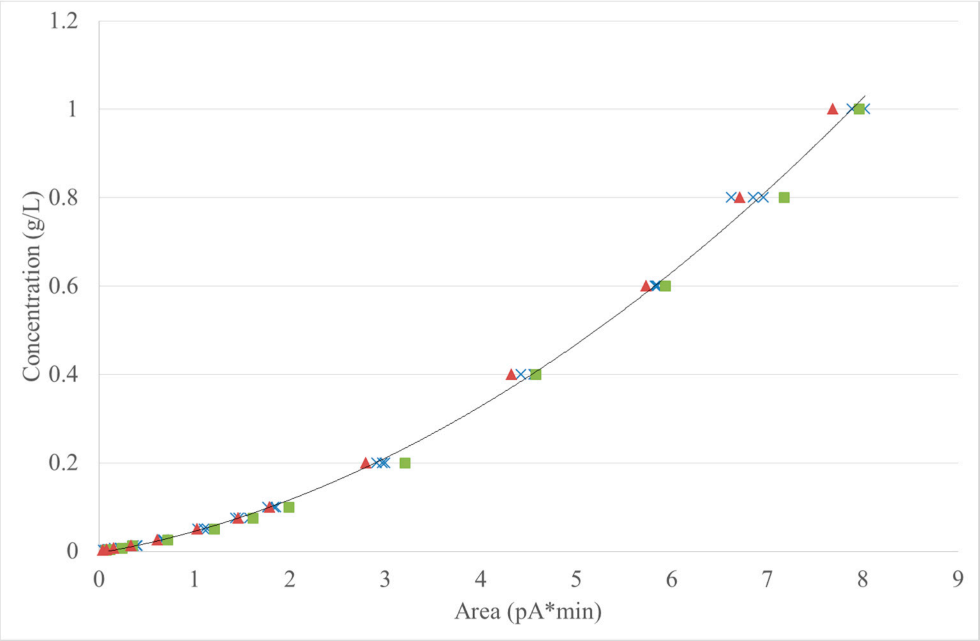

Figure S5. Calibration curve for glucose and effect of time on the response using RP-UHPLC-CAD. Triplicate standards analyzed within 24 h (blue crosses) with the corresponding quadratic fitting curve (solid line); standards analyzed one month later (green squares) and five months later (red triangles).

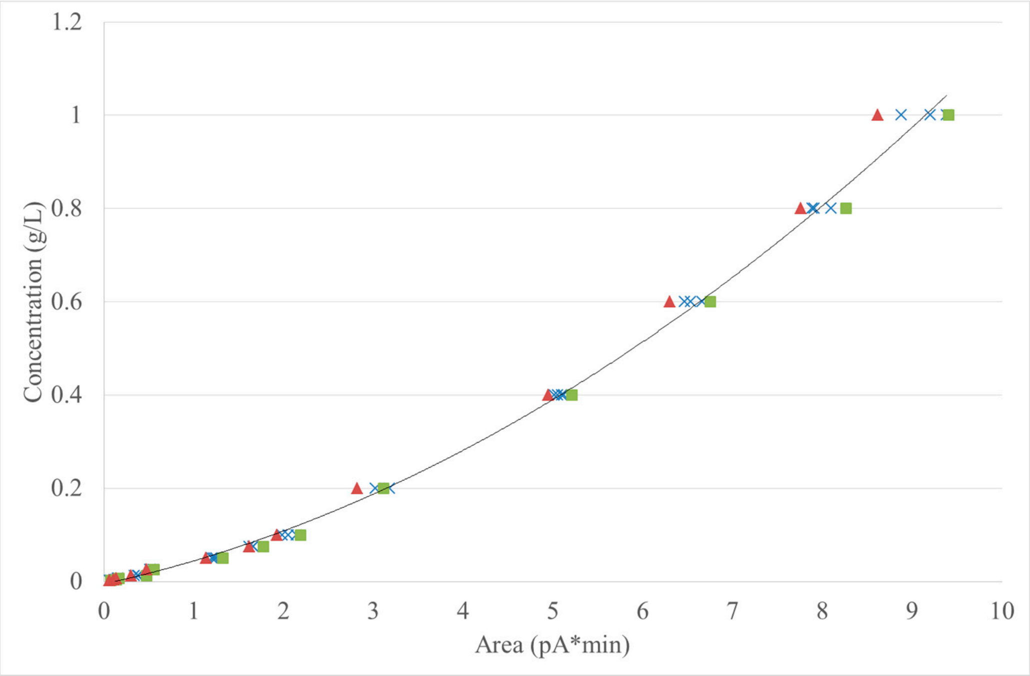

Figure S6. Calibration curve for xylose and effect of time on the response using RP-UHPLC-CAD. Triplicate standards analyzed within 24 h (blue crosses) with the corresponding quadratic fitting curve (solid line); standards analyzed one month later (red triangles) and five months later (green squares).

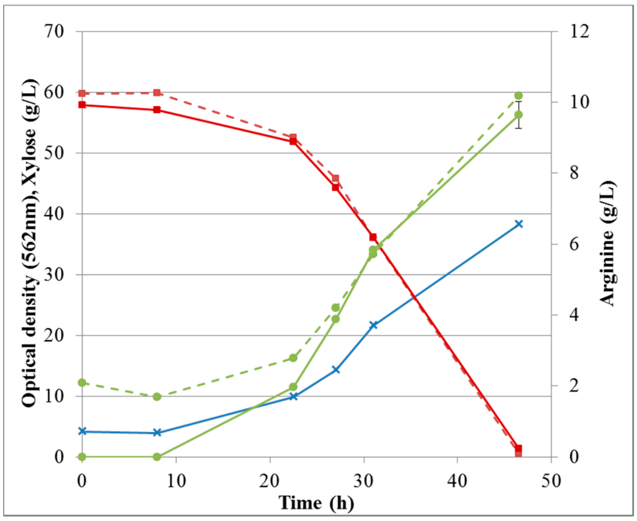

Figure S7. Fermentation profile: cell growth, xylose consumption, and arginine production in CSL. Optical density (blue crosses), xylose (red squares), and arginine (green circles) were determined using RP-UHPLC-CAD (solid lines) and HPAEC-IPAD (dashed lines). Standard deviations are omitted for clarity.
